# Poised for Allostery: Specific Interactions Underlie Precise Positioning of the C‑Terminal Tail in Phosphofructokinase‑1

**DOI:** 10.1021/acs.jpcb.6c03671

**Published:** 2026-10-26

**Authors:** Mehrnoosh Khodam Hazrati, Truong-An Nguyen, Stepan Timr

**Affiliations:** † 86875 J. Heyrovsky Institute of Physical Chemistry, Czech Academy of Sciences, Dolejskova 2155/3, 182 00 Prague 8, Czech Republic; ‡ Faculty of Science, Charles University, Albertov 6, 128 00 Prague 2, Czech Republic

## Abstract

Disordered terminal segments often regulate enzymes, but how such flexible elements achieve the spatial precision required for allostery remains poorly understood. Using human phosphofructokinase-1 as a model system, we combine enhanced-sampling molecular dynamics simulations, conservation-guided spatial analysis, prior allosteric network analysis, and coarse-grained modeling to show that allosteric positioning of the C-terminal tail, defined by residue-level contacts with the catalytic and regulatory domains, is maintained by a few key hydrogen bonding and hydrophobic interactions. This positioning enables the conserved tail residues L766 and E767 to dynamically couple the tail to the active site through a conserved surface region and to bridge the catalytic and regulatory domains in the T-state, experimentally associated with ATP inhibition. In contrast, coarse-grained simulations lacking explicit directional representation of hydrogen-bonding interactions fail to maintain stable tail docking, whereas including selected OLIVES-derived native hydrogen-bond terms improves the stability of the T-state-like docking pose. These findings support sequence-specific surface docking of a disordered enzyme tail as a physicochemical mechanism for allosteric regulation.

## Graphical abstract

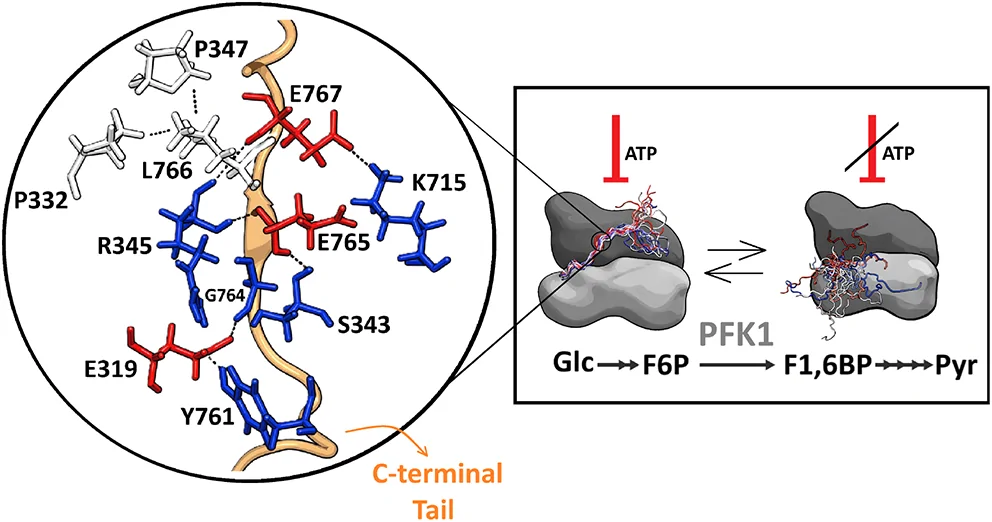

## Introduction

Disordered terminal segments are widespread regulatory elements that can modulate protein function through transient, multivalent, and often state-dependent interactions with structured protein surfaces.^1–3^ Their specificity is frequently encoded by short interaction-prone sequence elements, including molecular recognition features and short linear motifs, in which a small number of conserved residues form key contacts with a partner surface.^4,5^ For allosteric enzymes, this creates a physicochemical requirement: a flexible segment must retain conformational freedom while adopting a sufficiently precise surface-bound arrangement to stabilize or perturb a functional state.^6^ How residue-specific interactions generate such spatial precision, and to what extent this information can be retained in coarse-grained models, remain open questions.

Phosphofructokinase-1 (PFK1) provides a compelling system in which to examine this problem. PFK1 is a critical regulatory enzyme in glycolysis, the central metabolic pathway that converts glucose into pyruvate to produce energy in the form of ATP.^7,8^ As the enzyme catalyzing the rate-limiting step of glycolysis, PFK1 plays a key role in cellular energy homeostasis by controlling metabolic flux through this pathway. Its activity is tightly regulated by various allosteric effectors, such as ATP, ADP, AMP, and citrate, which ensure that energy production aligns with cellular needs.^9^ Dysregulation of PFK1 is implicated in numerous metabolic disorders, including cancer and diabetes, making it a major focus of biochemical and medical research.^10^

PFK1 is a large multisubunit enzyme with a complex quaternary structure that enables precise regulation.^11^ In humans, PFK1 exists as a tetramer (see Figure 1A) composed of tissue-specific subunits—muscle (PFKM), liver (PFKL), and platelet (PFKP)—which combine in different ratios depending on tissue type. Each subunit is composed of two domains—the catalytic and regulatory domains—that evolved from gene duplication and fusion. The subunit contains an active site for substrate binding and distinct allosteric sites for regulatory molecules. The enzyme transitions between active (R-state) and inactive (T-state) conformations in response to binding of allosteric activators, such as AMP, ADP, and fructose-2,6-bisphosphate, or inhibitors, such as ATP and citrate.^9^ These structural transitions allow PFK1 to fine-tune glycolytic flux in response to cellular energy demands. Advances in structural imaging^7,12,13^ have provided detailed insights into the three-dimensional architecture of the enzyme. Despite recent advances, the mechanisms of PFK1 regulation remain incompletely understood and are increasingly recognized as complex.^14^

**Figure 1:**
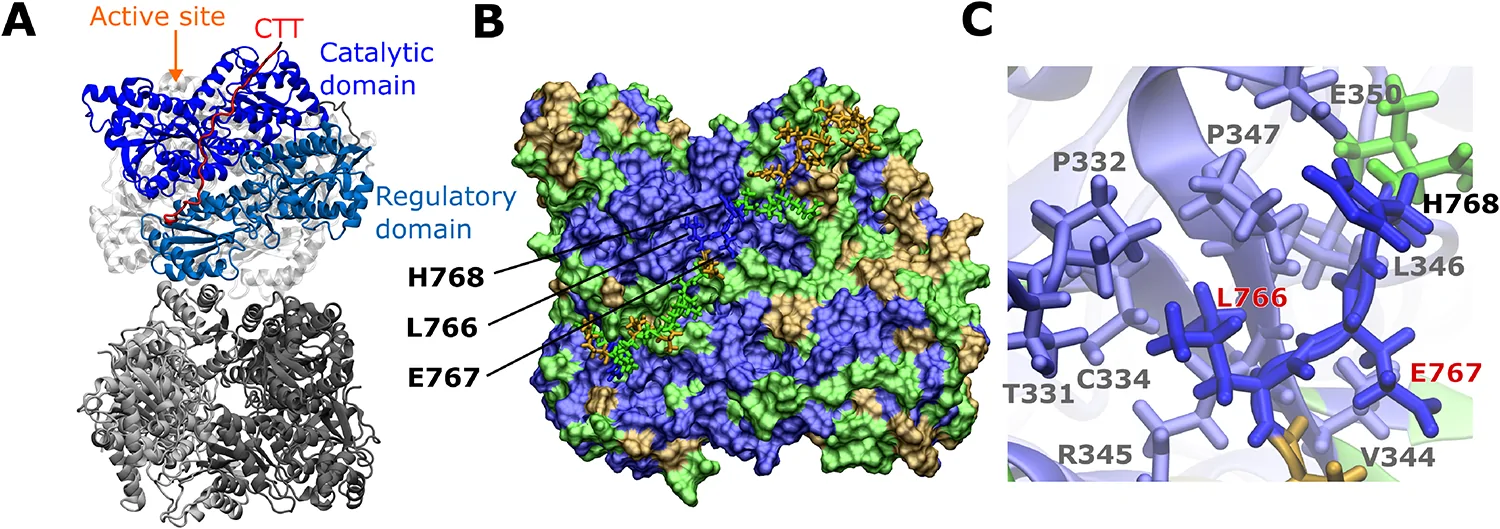
(A) Structure of the human PFKL tetramer based on the cryoEM structure (PDB ID: 8W2H),^13^ with CTT, catalytic and regulatory domains, and the active-site location highlighted in one subunit. The final nine residues of the CTT, which were unresolved in the experimental structure, were modeled as an unstructured segment. (B) PFKL subunit with residues colored by conservation across the three human PFK1 isoforms: conserved in all isoforms (blue), conserved in two isoforms (green), or not conserved (brown). (C) A detailed view of the conserved CTT residues L766, E767, and H768, and the surrounding conserved surface residues. CTT residues that are conserved across mammalian PFK1 isoforms^15^ are highlighted in red.

The C-terminal tail (CTT), which contains approximately 25 residues (Figure 1A), plays a significant role in PFK1 regulation. The CTT is indispensable for ATP-mediated allosteric inhibition, as CTT deletion suppresses the inhibitory effect of ATP in human PFK1 ^13,15^ and locks the enzyme in an activated state.^12^ Furthermore, phosphorylation of the CTT in response to innate immune signals increases PFKL activity and thereby promotes early glycolysis in macrophages.^16^ Recent cryoEM structures^13^ of human PFKL show that the CTT adopts an extended conformation bound to the surface of the PFKL T-state between the catalytic and regulatory domains. In contrast, the CTT conformation was unresolved in the active R-state, indicating that it becomes disordered upon PFKL activation.^13^ Our previous all-atom molecular dynamics (MD) simulations showed destabilization of the PFKL T-state conformation when the CTT was removed, and suggested that simultaneous binding of the CTT to both the catalytic and regulatory domains helps maintain their T-state-like relative angle.^13^ However, the simulations did not reach the time scales required to describe the conformational landscape of the CTT and its detachment from the enzyme surface.

In this work, we ask how the PFKL CTT achieves the spatial precision required for allosteric regulation. We combine all-atom replica-exchange umbrella sampling (REUS),^17^ Gaussian accelerated molecular dynamics (GaMD),^18,19^ conservation-guided spatial analysis, prior allosteric network analysis,^13^ and MARTINI 3 coarse-grained simulations^20^ to characterize state-dependent CTT conformational landscapes in the T- and R-states of PFKL. Apo monomers derived from T- and R-state structures were used as reduced structural proxies for the local CTT-binding surfaces of the corresponding full, ligand-bound enzyme states. The characterized CTT docking site is entirely intrasubunit, so monomerization removes none of the residues that directly form the docking interface. Moreover, the restraints applied in the monomer simulations maintain the subunit close to its tetramer-derived conformation, thereby preserving the local geometry of the docking surface. Our results support an interdomain bridging arrangement of the CTT in the inhibited T-state and show that this allosterically relevant positioning is stabilized by a small set of residue-specific hydrogen-bonding and hydrophobic interactions with the catalytic-domain surface. In contrast, coarse-grained simulations lacking explicit representation of hydrogen-bonding interactions lead to loss of allosterically competent CTT positioning.

The simulations were designed to resolve the local structural determinants of CTT docking rather than to compare ligand-occupancy conditions or reproduce the complete tetrameric regulatory response. Their functional interpretation is supported by previous cryoEM structures showing a well-defined surface-bound CTT in the T-state,^13^ biochemical evidence that CTT truncation or mutation of L766 and E767 reduces ATP inhibition,^12,13,15^ and allosteric network analysis linking these residues to the active site.^13^ The present simulations identify the residue-level interactions that maintain this allosteric positioning, but do not directly quantify how ligand occupancy modifies the interaction network.

## Methods

### Initial Protein Conformations

Interactions between the C-terminal tail (CTT), formed by residues 753–780, and the surface of the human liver PFK1 (PFKL) were investigated by simulating apo-state PFKL monomers extracted as individual subunits from experimentally obtained tetrameric structures of PFKL. Four distinct systems were prepared: one to characterize CTT behavior in the T-state and three to explore CTT behavior in the R-state (R­(transposed), R­(transition 1) and R­(transition 2)); see Table 1.

**Table 1:** Summary of the Structural Origins of the R-state-like Systems

System	Subunit source	CTT source
R(transition 1)	Biased T-to-R simulation	Biased T-to-R simulation
R(transition 2)	Biased T-to-R simulation	Biased T-to-R simulation
R(transposed)	R-state cryoEM	T-state cryoEM

#### T-state System

The T-state system was based on the same initial protein structure that was used in our previous work.^13^ Namely, the model was built using a monomer extracted from the cryoEM structure of human PFKL in the T-state (PDB ID: 8W2H), with the last nine C-terminal tail residues, which were not resolved in the experiments, modeled using the PyMOL 2.5.4 software.^21^ The first 10 residues of the N-terminus, which were also missing from the cryoEM structure, were not included in our simulations. The protonation states of histidine residues were adopted from the deposited PDB structure, which contains explicit hydrogen atoms.

#### R­(transposed)

The first initial R-state protein structure was adopted from our previous work.^13^ To construct this model, the T-state structure described above was first equilibrated using the protocol in Table S1. The equilibrated T-state model was then superposed onto the R-state cryoEM structure (PDB ID: 8W2I) by RMSD alignment over residues 13–752. After alignment, residues 13–752 were retained from the R-state structure, whereas the CTT coordinates (residues 753–780) were retained from the equilibrated T-state model. The resulting R­(transposed) model was subsequently minimized and equilibrated for the REUS and GaMD calculations using the method-specific protocols described below. The first 12 N-terminal protein residues, not resolved in the cryoEM structure, were not included. The protonation states of histidine residues were adopted from the respective deposited PDB structures.

#### R­(transition 1) and R­(transition 2)

Two alternative conformations of the R-state were obtained from a biased molecular dynamics simulation, in which a T-to-R conformational transition was induced. For computational efficiency, the conformational transition was simulated in a PFKL dimer rather than the full tetramer. The initial conformation of the T-state dimer was taken from our previous work,^13^ as described above for the T-state monomer. The T-to-R conformational transition and a subsequent relaxation were performed in the presence of ligands bound to the T-state conformation (three ATP molecules, one F1,6BP molecule, and one Mg^2+^ ion for each of the two subunits). The system was placed in a rhombic dodecahedral simulation box with the nearest-image distance of 15.3 nm and solvated by 74593 water molecules. 253 K^+^ ions and 227 Cl^–^ ions were added to the simulation box to charge-neutralize the system and to achieve the physiological ionic strength of 150 mM. The CHARMM36m force field^22^ was employed to parametrize the protein; water molecules were described with the TIP3P water model,^23^ and default ion parameters associated with CHARMM36m were used. ATP molecules were described using existing CHARMM parameters obtained from the CHARMM-GUI Web server,^24,25^ and F1,6BP molecules were parametrized by CHARMM-GUI using the CHARMM General Force Field (CGenFF).^26^

The biased MD simulation of the T-to-R conformational transition was performed using GROMACS 2022.5 ^27,28^ patched with PLUMED 2.8.2.^29,30^ The biasing coordinate was defined in PLUMED using CONTACTMAP with CMDIST, based on Cα–Cα contacts that differ between the experimentally resolved PFKL T- and R-state structures. Specifically, only those residue pairs were included for which the Cα–Cα distance was below the native-contact cutoff of 10 Å in one structure but not in the other. Residue pairs for which the Cα–Cα distance fluctuated within 2 Å of the cutoff were excluded. In total, 436 residue pairs were included in the evaluation of the biasing coordinate.

Each selected pair distance was transformed into a contact-map component using a rational switching function (*r*_0_ = 10 Å, *d*_0_ = 0, *n* = 6, *m* = 12), as implemented in PLUMED, and the collective variable was defined as the distance from the reference contact map of the target state. First, the system was energy-minimized and equilibrated in the absence of bias along the collective variable (see Table S1 for details on the six-step equilibration protocol). Subsequently, the transition toward the R-state was induced by applying a one-sided harmonic potential centered at 0.0 on the contact-map distance, while ramping the force constant linearly from 0 to 20 kJ·mol^–1^ over 320 ns. This was followed by a 2 μs unbiased relaxation.

During both the transition and the subsequent relaxation, the system was propagated in the NPT ensemble with a 4 fs time step, enabled by the hydrogen mass repartitioning (HMR) approach.^31^ Bonds involving hydrogen atoms were constrained using LINCS.^32^ Temperature was maintained at 300 K using separate velocity-rescaling thermostats for solute and solvent (τ = 0.1 ps),^33^ and pressure was controlled isotropically at 1.01 bar using a Parrinello–Rahman barostat (τ = 1.0 ps).^34^ Short-range electrostatic and van der Waals interactions were truncated at 1.2 nm, and long-range electrostatics were treated using the particle-mesh Ewald (PME) method,^35^ using a grid spacing of 1.2 nm, a real space cutoff of 1.2 nm, and PME order 4.

The resulting conformations of the two subunits were termed R­(transition 1) and R­(transition 2), respectively.

### Correlation Network Analysis

The network results in Figure 2 were obtained from three previously reported 400 ns simulations of full-length, ligand-bound T-state PFKL tetramers, with the first 50 ns of each trajectory excluded from the analysis, as described in ref 13. Specifically, ensemble correlation network analysis^36^ was performed with Bio3D using residues represented by their C_α_ atoms. After mass-weighted superposition of each subunit together with its neighboring subunit across the catalytic interface, residue cross-correlations (*c*_*ij*_) were calculated separately for each subunit and trajectory. A consensus edge was retained when |*c*_*ij*_| ≥ 0.5 in all subunits and trajectories, or when |*c*_*ij*_| ≥ 0.5 in at least one subunit and trajectory and the corresponding C_α_ atoms remained within 10 Å for at least 75% of all analyzed frames; edge weights were determined from the retained correlation values. For Figure 2A, each CTT residue was paired with R210, and 500 optimal and suboptimal paths were generated for each end point pair. Shorter path lengths, with the path length being defined as the sum of edge weights *w*_*ij*_ = −ln­(|*c*_*ij*_|), indicate stronger dynamic coupling; the line in Figure 2A reports the shortest path, and the shaded region spans the range of suboptimal path lengths.

**Figure 2:**
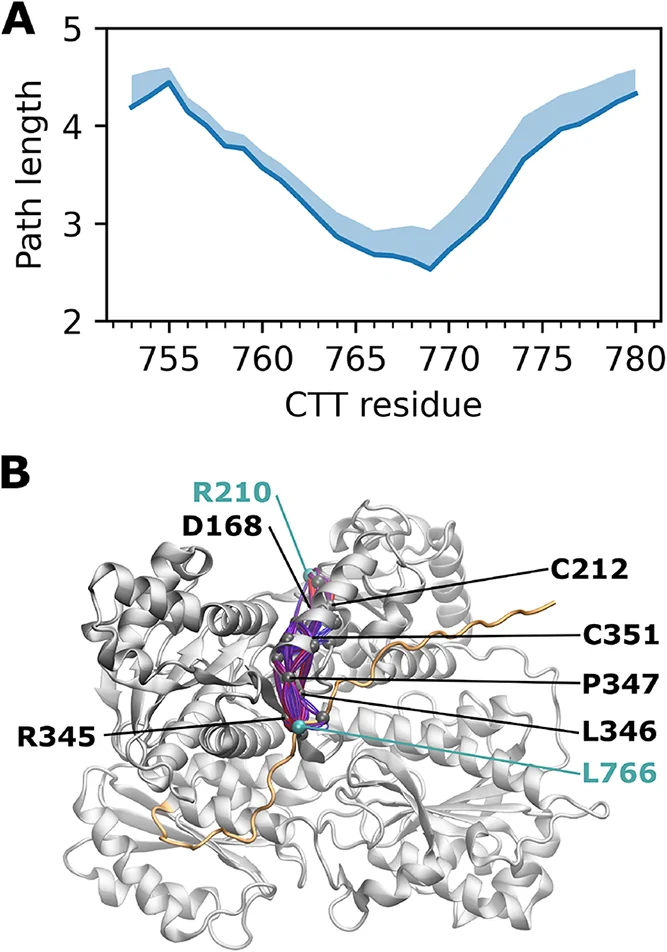
(**A**) Allosteric network analysis^13^ shows that the conserved CTT residues L766 and E767 are tightly coupled to the active-site residue R210, playing an important role in the coordination of the substrates. (**B**) An ensemble of shortest paths coupling residue L766 to the active-site residue R210. The paths cross residues L346 and P347 from the conserved hydrophobic region on the enzyme surface.

### Replica-Exchange Umbrella Sampling Simulations

Replica-exchange umbrella sampling (REUS)^37^ was employed to investigate the T- and R-state conformations of PFK1 monomers. Prior to simulation setup, all solvent molecules, ions, and bound ligands were removed from the cryoEM PDB structures to retain only the protein coordinates for subsequent modeling and simulation procedures. The T-, the R­(transposed), and the R­(transition 2) monomers were each placed separately in a rhombic dodecahedral simulation box with the nearest-image distance of 15.5 nm, and periodic boundary conditions were applied in all directions. Following the solvation process, the system was supplemented with K^+^ and Cl^–^ ions at physiological concentrations of 150 mM. The systems were minimized and equilibrated using the same protocol as in Table S1. Simulations were performed using the identical MD software, force field, and general simulation settings as described in the T-to-R conformational transition, unless noted otherwise.

A distance restraint matrix was imposed for Cα pairs (excluding the CTT) within a cutoff distance of 0.8 nm using GROMACS bond type 10 potentials, with a harmonic force constant of 1000 kJ·mol^–1^·nm^–2^. This approach maintains overall conformational state in either the T or R throughout the simulations while allowing local structural fluctuations.

The collective variable (CV) was defined as the projection of the center of mass (COM) of Q753–R771 of the C-terminal residues along the axis defined by the COM of two anchor points: the C atoms of the carbonyl groups of V335 and R345. The PROJECTION_ON_AXIS command from the colvar module of PLUMED 2.8.2 ^29,30^ was used to calculate the positions based on projection along and extension from the defined axis. This CV was used to monitor conformational changes during the simulations. The CTT segment was pulled along the CV using MOVINGRESTRAINT in the bias module of PLUMED 2.8.2 ^29,30^ with a harmonic potential force constant of 500 kJ·mol^–1^·nm^–2^ during 10000000 steps. Subsequently, REUS simulations were performed for 64 windows equally spaced by 0.07 nm covering the range from 0.0 to 4.41 nm, with each window simulated for 200 ns. This window spacing and simulation length were selected to ensure sufficiently high exchange probabilities >0.2. The convergence analysis is reported in Figure S1. The CV restraint forces and bias potentials were recorded every 20 simulation steps in COLVAR files. Postsimulation, we followed the Münster tutorial by Bonomi and Bussi available online as part of the PLUMED Tutorials collection^38^ to reconstruct the free energy landscape along the CV from the second half of the trajectories using the WHAM method.^39^ Statistical uncertainties were estimated by bootstrap analysis using 200 bins to discretize the reaction coordinate.

To further characterize the CTT unbinding process, we performed a standard reweighting analysis to project the free-energy profiles onto the fraction of native contacts (*Q*_*N*_). The native contacts were defined for the CTT residues 753–771, excluding the covalent contact involving residues 752–753, resulting in a total of 46 native contacts (see Figure S2). Each contact was assigned an equal weight of 1/46. The contacts were evaluated using the PLUMED CONTACTMAP function with a switching function characterized by *R*_0_ = 0.01 nm, β = 50.0, and LAMBDA = 1.5; the native reference distance for each contact was determined from the T-state CryoEM structure. *Q*_*N*_ was evaluated using the same contact definition for all three systems analyzed: T, R­(transposed), and R­(transition 2). The native-contact coordinate was calculated in postprocessing using the PLUMED driver from the second half of the combined REUS trajectories. The trajectories from the 64 REUS windows were analyzed simultaneously using --multi 64, while the original umbrella coordinate and restraint potential were retained in the PLUMED input to identify the corresponding bias energies. The resulting *Q*_*N*_ values were then combined with the WHAM-derived statistical weights obtained from the REUS simulations to reweight the configurations and construct the free-energy profiles as a function of *Q*_*N*_ (see Figure S2). This analysis was performed independently for each of the three systems to assess whether additional intermediates could be resolved along the native-contact coordinate.

### Gaussian Accelerated Molecular Dynamics (GaMD) Simulations

The Gaussian Accelerated Molecular Dynamics (GaMD) approach^18^ implemented in AMBER 2022 ^40^ was carried out using the AMBER ff14SB force field,^41^ and the TIP3P water model.^42^ The PFKL T and R­(transposed) monomers were built using the tLEaP module of the AMBER package.^40^ The systems were solvated in a TIP3P water box, extending 15 Å from the solute surface. Counterions (Cl^–^ and Na^+^) were randomly added to establish a 150 mM ionic strength. Periodic boundary conditions were applied for the simulation systems. In all simulations, positional restraints of 10 kcal·mol^–1^·Å^–2^ via a flat-bottom potential on the backbone atoms of the protein, excluding the CTT, were applied to maintain the configurations in T- or R-state.

Water and ions were initially minimized for 20,000 steps with protein heavy-atoms harmonically restrained with a force constant of 10 kcal·mol^–1^·Å^–2^. Then, to relax the whole system, the restraints were removed and 2000 steps of minimization under a nonbonded cutoff of 10 Å and the SHAKE algorithm^43,44^ was performed. The system was heated from 0 to 300 K over 1 ns under an isothermal-isovolumetric (NVT) condition with positional restraints of 20 kcal·mol^–1^·Å^–2^ on all protein non-hydrogen atoms. The temperature was controlled through the Andersen thermostat^45^ with a collision frequency of 0.5 ps^–1^. The restraint weights were ramped via NMR-style input to release the constraints smoothly. To allow water penetration into protein cavities, a 40 ps unconstrained MD was performed in an isothermal–isobaric (NPT) ensemble with a coupling constant of 1.0 ps at 300 K with protein restraints maintained at 10 kcal·mol^–1^·Å^–2^. Long-range electrostatic interactions were treated with the Particle Mesh Ewald (PME) method,^35^ using a cutoff of 10 Å.

The systems were first equilibrated for 500 ps in an NPT run with positional restraints of 10 kcal·mol^–1^·Å^–2^ on the heavy atoms of the protein using the Langevin thermostat^46^ with γ = 2 ps^–1^ and a Monte Carlo barostat^47^ with a coupling constant of 2 ps. Then it was followed by a 5 ns NPT equilibration and 2 ns conventional MD (cMD) production runs without any restraints at 300 K and 1 atm, using the Langevin thermostat^46^ with γ = 2 ps^–1^ and an isotropic Berendsen barostat^48^ with a coupling constant of 1 ps. The hydrogen-mass repartitioning approach^49^ allowed for a 4 fs integration time step.

Using the PPI-GaMD approach,^19^ we enhanced sampling of the interactions between the CTT and the rest of the protein in the T- and R-state systems. The PPI-GaMD simulations proceeded with a 22 ns short cMD to collect the potential statistics, followed by a 100 ns PPI-GaMD equilibration after adding the boost potentials, and then 2 μs production runs. Because the PPI-GaMD implementation in AMBER 22 did not support the Monte Carlo barostat, the isotropic Berendsen barostat described above was retained during all three stages, including the 2 μs production runs. The simulation was repeated for three replicas. We applied dual boosting (*igamd* = 17), in which the boost potential was applied to the dihedral energy and the total nonbonded interaction potential energy between the CTT and the rest of the protein. The upper limit of the standard deviation of the first and second boost potentials was set to 2.0 kcal·mol^–1^.

The 2D histograms of distances were generated for the distance between the centers of mass of carbon atoms of residues P347 and L766, and the distance between the K715:NZ and E767:CD atoms (SB1). Additional 2D histograms were constructed for the shorter of the two distances between E319:OE1/OE2 and G764:HN, together with the distance between R345:O and E767:HN (HB1). Furthermore, 2D histograms of the distance between the centers of mass of carbon atoms of residues M354 and V769, and the distance between the centers of mass of carbon atoms of residues K677 and F780 were generated. The resulting histograms are shown in Figure S3. All distance analyses were performed using the CPPTRAJ module^50^ of AMBER 22 ^40^ by combining trajectories from three independent 2 μs replicas, with frames saved every 1 ns, yielding a total of 6000 frames. Distance-versus-time plots for each replica of the T-state and R­(transposed) are presented in Figures S4 and S5, respectively.

### MARTINI 3 Coarse-grained Simulations

We performed unbiased simulations for the T and R­(transition 2) using the standard MARTINI 3 (v3.0.0)^20^ and scaled MARTINI 3, in which we rescaled protein–protein interactions by λ_PP_ = 0.88, suggested by Thomasen et al.^51^ Martinize2 ^52^ was used to convert atomic structures into coarse-grained MARTINI structures and topologies. The coarse-grained T- or R-state monomers were simulated using the GROMACS version 2023 software package.^28,53^ An elastic network (EN) was applied to the backbone beads of the enzyme (excluding CTT residues) with 700 kJ·mol^–1^·nm^–2^ harmonic force constant.

The T and R­(transition 2) monomers were each placed separately in a rhombic dodecahedral simulation box with the nearest-image distance of 13.22 and 13.75 nm, respectively. The T-state system was solvated with 11570 water molecules, whereas the R­(transition 2) system contained 13170 water molecules. After solvation, the systems were neutralized and supplemented to a physiological salt concentration of 150 mM by adding 148 Na^+^ and 149 Cl^–^ ions to the T-state system, and 166 Na^+^ and 167 Cl^–^ ions to the R­(transition 2) system. Additional Cl^–^ ions were added to neutralize the net charge. The systems were energy-minimized and equilibrated through a 10 ns NPT equilibration run with a 20 fs integration time step. Reaction-field electrostatics was applied with a cutoff of 1.2 nm and ϵ_r_ = 15. The van der Waals interactions were calculated using the potential-shift Verlet cutoff-scheme with a cutoff of 1.2 nm. Temperature and pressure were maintained at 300 K and 1 bar using the v-rescaling thermostat^33^ and the c-rescale barostat,^54^ with coupling constants of 1 and 12 ps, respectively. For production runs, the Parrinello–Rahman barostat^34^ was used with a coupling constant of 12 ps and a compressibility of 3e-4 bar^–1^. The periodic boundary conditions were applied in all directions, and the integration time step was set to 20 fs. Production molecular dynamics (MD) runs were conducted for 25 μs. The simulations were repeated for three independent replicas with different initial velocities for each system.

Since the MARTINI 3 simulations indicated reduced stability of the bound CTT in the T-state, we repeated the simulations only for the T-state system by incorporating specific interaction terms^55^ to test whether explicit representation of the identified T-state β-strand hydrogen bonds could maintain the T-state-like CTT docking pose. Specifically, we described the hydrogen-bonding interactions between backbone beads of R345–E767, R345–E765, and S343–E765 using the corresponding OLIVES LJ intermolecular parameter, σ = 0.3920987698 nm, and ϵ = 26.82096 kJ·mol^–1^.^55^ All other simulation parameters were identical to those used in the standard MARTINI 3 simulations. Production MD runs were then carried out for 15 μs across three independent replicas with different initial velocities.

## Results and Discussion

### Precise CTT Positioning is Required for its Allosteric Role

Sequence analysis (see Figure 1B) shows that approximately 70% of residues are conserved among the three human PFK1 isoforms, and buried residues tend to be conserved more than surface residues. In the CTT, most residues are not conserved among human isoforms, except for Y754, L766, E767, and H768 (Figure 1B). Although H768 is conserved among the three human PFK1 isoforms (see Figure 1C), it is not conserved across mammalian PFK1 sequences, unlike L766 and E767.^15^ We therefore focus on L766 and E767, mutations of which were previously shown to abolish the sensitivity of PFK1 to inhibition by ATP.^15^

The residues L766 and E767 are located near a conserved hydrophobic patch on the enzyme surface (Figure 1B). This patch is formed by two symmetrically positioned prolines, P332 and P347, as well as hydrophobic residues C334, V344, and L346, and hydrophilic residues T331, R345, and E350. The conservation of this three-residue CTT segment, located near another conserved surface region, strongly suggests that the spatial proximity of these regions is functionally important.

Allosteric network analysis of our previously published T-state PFKL trajectories^13^ identified optimal and suboptimal network paths between selected source and sink residues.^36^ These network paths highlight residues involved in the dynamic coupling of the two end points. The length of a path is inversely proportional to the strength of the coupling. The analysis reveals that the conserved CTT residues L766 and E767 are part of the CTT segment most strongly coupled to the active site residue R210 (see Figure 2A). This residue, whose geometry changes between the T- and R-states, plays an important role in substrate coordination.^13^ Furthermore, the network paths traverse the conserved region on the enzyme surface, including residues L346 and P347 (Figure 2B). This indicates that the conserved surface region is dynamically sensitive to the presence of the CTT and that this sensitivity is transmitted to the active site, potentially affecting PFKL activation. To determine whether the T-to-R transition disrupts the proposed communication pathway beyond displacement of the CTT, we examined changes in native contacts along the L766–R210 suboptimal paths. No altered contact connected two residues on these paths (Figure S6), suggesting that the portion of the pathway within the catalytic domain remains structurally continuous in the R-state, although some path residues, including R210, changed contacts with residues outside the paths.

### CTT Forms a Bridge Between Domains in T-State but Not in R-State

Standard MD simulations are inherently limited by the time scales they can cover. Therefore, to more thoroughly characterize the CTT binding in the T-state, we performed an enhanced-sampling REUS simulation^17^ of gradual CTT displacement from the enzyme surface. CTT unbinding was driven along a collective variable defined as the projection of the center-of-mass position of the bound CTT segment (residues 753–771) onto an axis approximately perpendicular to the enzyme surface (see Methods). The REUS simulation consisted of 64 windows, each simulated for 200 ns. Figure S7 shows that the CTT docking surface and adjacent regions retain their conformation found in the T-state cryoEM structure during the REUS simulation.

The simulation confirms that at the free-energy minimum, corresponding to the native binding position, L766 engages in hydrophobic interactions with P347 in the conserved region on the surface of PFKL (see Figure 3A,B). At the same time, E767 forms a salt bridge with residue K715 of the regulatory domain (Figure 3A,B). At this position, near the entrance of the nucleotide activating site, all human PFK1 isoforms contain a positively charged residue. Substitution by arginine in the muscle and platelet isoforms may further stabilize this interaction by enabling a stronger salt bridge.

**Figure 3:**
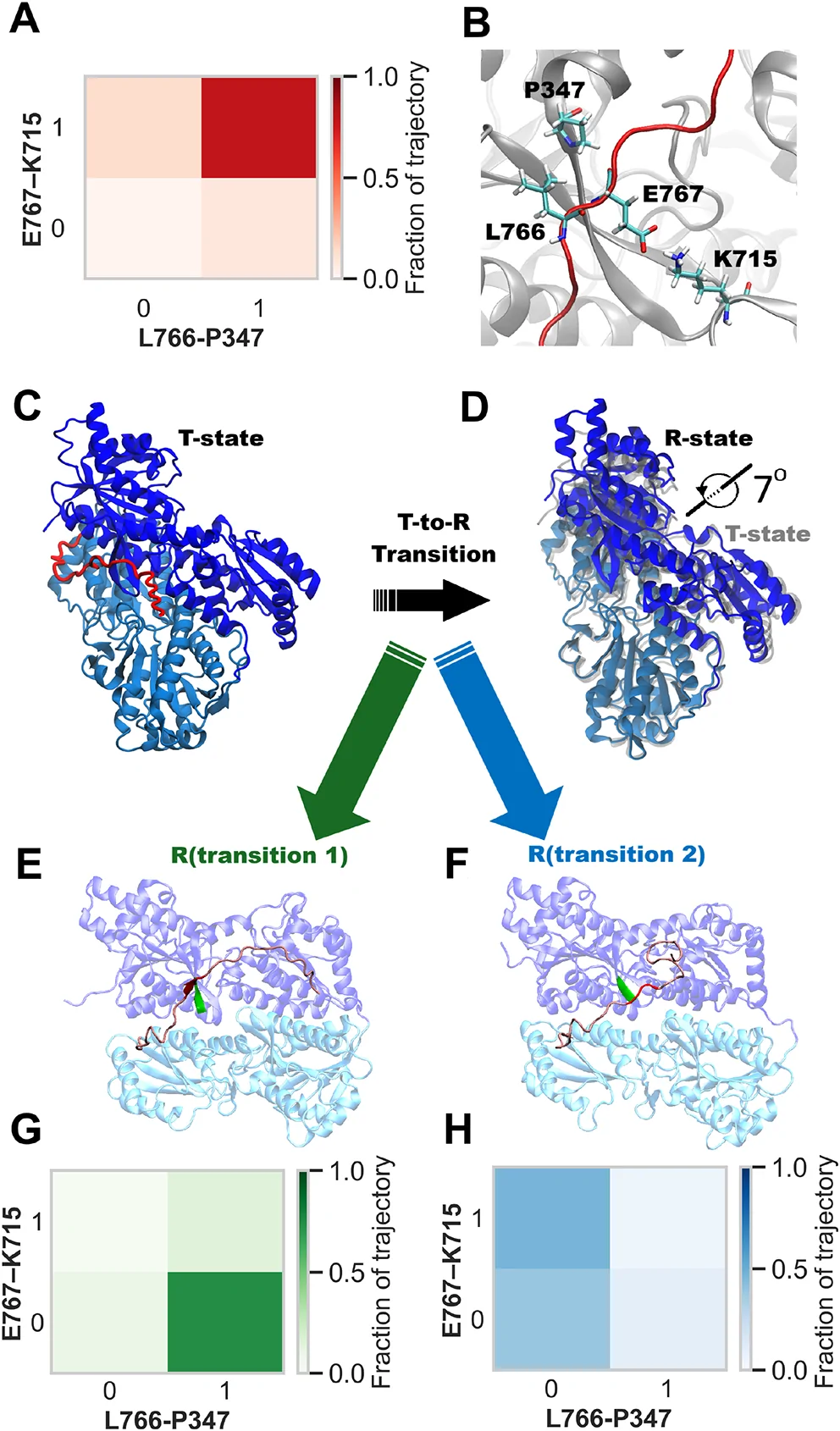
Bridging role of the CTT in the T-state. (A) The fraction of the trajectory during which the L766–P347 and E767–K715 contacts were observed. A value of 1 means that the pairs were in contact throughout the simulation, while a value of 0 means no contact. In the T-state, the conserved CTT residue L766 forms hydrophobic contacts with P347 from the adjacent conserved region on the enzyme surface. At the same time, the conserved CTT residue E767 engages in a salt bridge with K715 from the regulatory domain. The values were obtained from the second half of the trajectory corresponding to the REUS window at the minimum of the free-energy profile. (B) Representative geometry showing L766 and E767 forming the interdomain bridge. (C) T-state monomer and (D) R-state monomer. (E) R­(transition 1) and (F) R­(transition 2), two outcomes of the T-to-R transition. The presence of L766–P347 contacts and E767–K715 salt bridge for R­(transition 1) (G) and R­(transition 2) (H). The values were obtained from a 2 μs relaxation trajectory following the T-to-R transition. A contact cutoff of 0.5 nm was used between carbon atoms of P347 and L766 and a contact cutoff of 0.7 nm between the K715:NZ and E767:CD atoms.

The structural transition between the inactive T-state and the active R-state involves domain rotation and rearrangements (see Figure 3C,D).^13^ To investigate how interactions of the CTT with the enzyme surface evolve upon the conformational transition from T to R, we induced the T-to-R transition in a T-state dimer using a biased MD simulation protocol (Figure S8A,B). The transition, followed by a subsequent 2 μs relaxation (Figure S8C,D), resulted in a distinct CTT conformation in each subunit, hereafter referred to as R­(transition 1) and R­(transition 2), respectively (Figure 3C–F). Domain-angle and contact-map-distance analyses confirm movement toward R-state-like global geometry, while local C_α_ RMSDs indicate residual deviations at the CTT-docking surface after relaxation (Figure S9). Thus, R­(transition 1) and R­(transition 2) are used as R-like mechanistic comparators, although their local CTT-docking-surface geometries may differ from the experimental R-state. The conformations obtained after relaxation for R­(transition 1) and R­(transition 2) (see Figure 3E and F, respectively) show that while the CTT retained an approximately T-state-like conformation on the surface of the catalytic domain in R­(transition 1), its C-terminal part became displaced in R­(transition 2) (Figure 3F). In both cases, we observed loss of CTT-mediated bridging, as indicated by the interaction pairs P347–L766 and K715–E767 (see Figure 3G,H; the time evolution of these contacts during the preceding T-to-R transition and relaxation trajectories can be found in Figure S10). Specifically, the salt bridge of K715–E767 was broken for R­(transition 1), while the hydrophobic interaction of P347–L766 was disrupted in R­(transition 2). This highlights that, due to the change in the domain angle associated with the T-to-R transition, both bridging interactions are unlikely to be formed simultaneously.

During the relaxation trajectory following the T-to-R transition, we also observed an increase in both the average distance between P332 and P347 and its fluctuations in the subunit with the fewest P347–L766 contacts (see Figure S11). P332 and P347 are located on the N-terminal subdomain of the catalytic domain and on the two-helix hinge coupling the catalytic domain with the regulatory domain, respectively. Therefore, the relative motions of these two highly conserved residues may correlate with larger-scale PFK1 conformational rearrangements and propagate these effects to the active site. By amplifying motions of P332 and P347, CTT unbinding might potentiate the conformational flexibility of active site residues required for catalytic activity; however, the present analysis does not directly establish coupling of the P332–P347 distance to R210 or other active-site residues.

### Proper Positioning of CTT Enabled by a Short β-Strand Motif

In the native bound position of the CTT in the T-state, our REUS simulation revealed a short β-strand motif formed by CTT residues 765–767 (see Figure 4A and Tables S2 and S3). This β-strand connects the CTT to the surface of the catalytic domain through three backbone hydrogen bonds (HB1–HB3) involving CTT residues E765 and E767 and catalytic-domain residues S343 and R345 (see Figure 4A).

**Figure 4:**
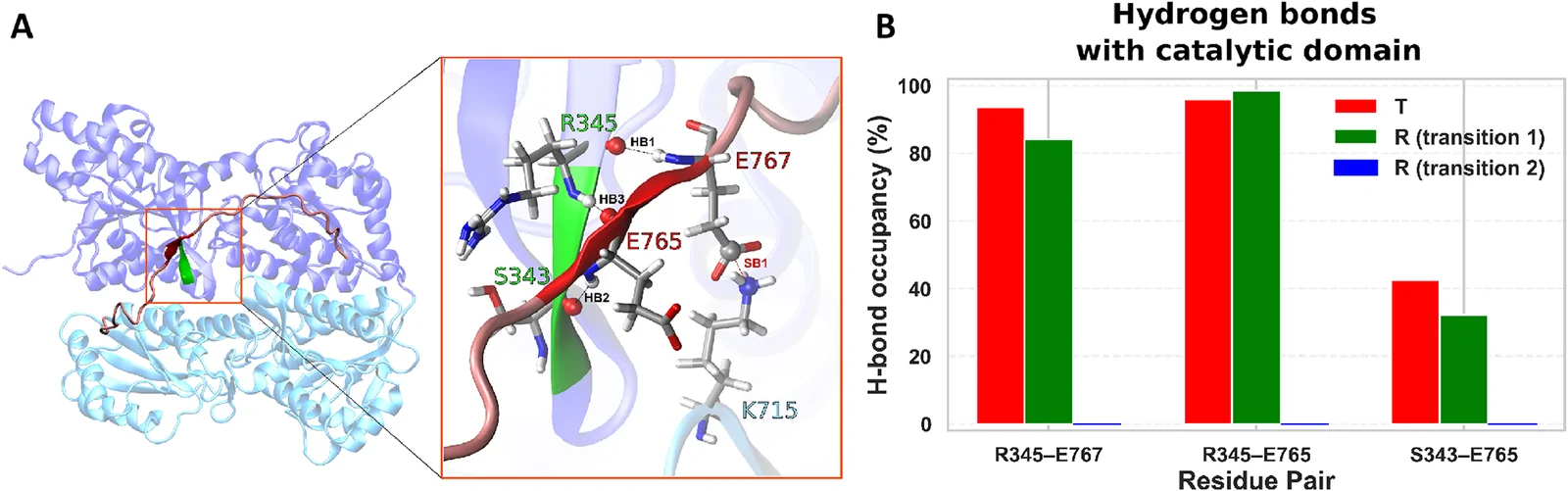
(A) Short β-strand motif formed by CTT residues 765–767 with the surface of the catalytic domain maintains the proper positioning of this key CTT segment with respect to the catalytic domain. (B) Existence of hydrogen bonds forming the β-strand in the T-state, as observed in the minimum free-energy window of the REUS simulation over the second half of the trajectory, and in the two outcomes of the simulated T-to-R transition during a subsequent 2 μs relaxation trajectory. While these hydrogen bonds are present in the T-state, they are disrupted during the T-to-R transition in R­(transition 2). **HB1:** R345:O–E767:HN; **HB2:** R345:HN–E765:O; **HB3:** S343:O–E765:HN; **SB1:** K715:NZ–E767:CD.

We found that this β-strand motif helps maintain the position of the C-terminal portion of the surface-bound CTT segment near the conserved region on the surface of the catalytic domain while allowing E767 to interact with K715 of the regulatory domain. As shown in Figure 4B, breaking the motif during the T-to-R transition correlates with the displacement of the terminal part of the bound CTT in R­(transition 2) (see Figure 3F).

### Further Important Interactions Stabilizing CTT Binding

Our REUS simulations^17^ of CTT detachment from the enzyme surface allowed us to monitor interactions of the CTT and obtain the free-energy profile along a collective variable tracking the detachment. Figure 5A,B show the evolution of hydrogen-bonding and hydrophobic interactions stabilizing the native bound structure of the CTT (see Methods) along the detachment collective variable. The interacting residues can be divided into three main regions: a hydrophobic patch involving residues 756–760 at the N-terminal end of the CTT, a middle region involving hydrogen bonds primarily grouped around the conserved residue E319, and a terminal region featuring the β-strand hydrogen bonds described above as well as hydrophobic interactions with the conserved patch on the protein surface. The free-energy profile (Figure 5C) shows that the strong increase in free energy when leaving the native binding position is associated with the breaking of the interactions in the latter two regions. This ultimately leads to a displacement of the CTT from the enzyme surface. Therefore, these interaction centers play an important role in CTT stabilization. It should be noted that these profiles are biased-coordinate projections of high-dimensional free-energy surfaces onto a single CTT-displacement coordinate on restrained T- or R-like protein surfaces, rather than physical unbinding free energies of unconstrained PFKL. Incomplete equilibration of orthogonal degrees of freedom within individual umbrella windows may limit sampling of conformational entropy, especially for the flexible C-terminal tail, and may therefore overestimate the thermodynamic cost of detachment. Accordingly, the absolute barriers and end-point values, as well as differences among the profiles, are not interpreted as quantitative unbinding thermodynamics. Instead, when considered together with the window-resolved interaction analyses in Figure 5A,B and Figures S12A,B and S13A,B, the profiles identify where native contacts are lost along the enforced detachment paths and implicate the central and terminal contact regions in maintaining the native docking pose.

**Figure 5:**
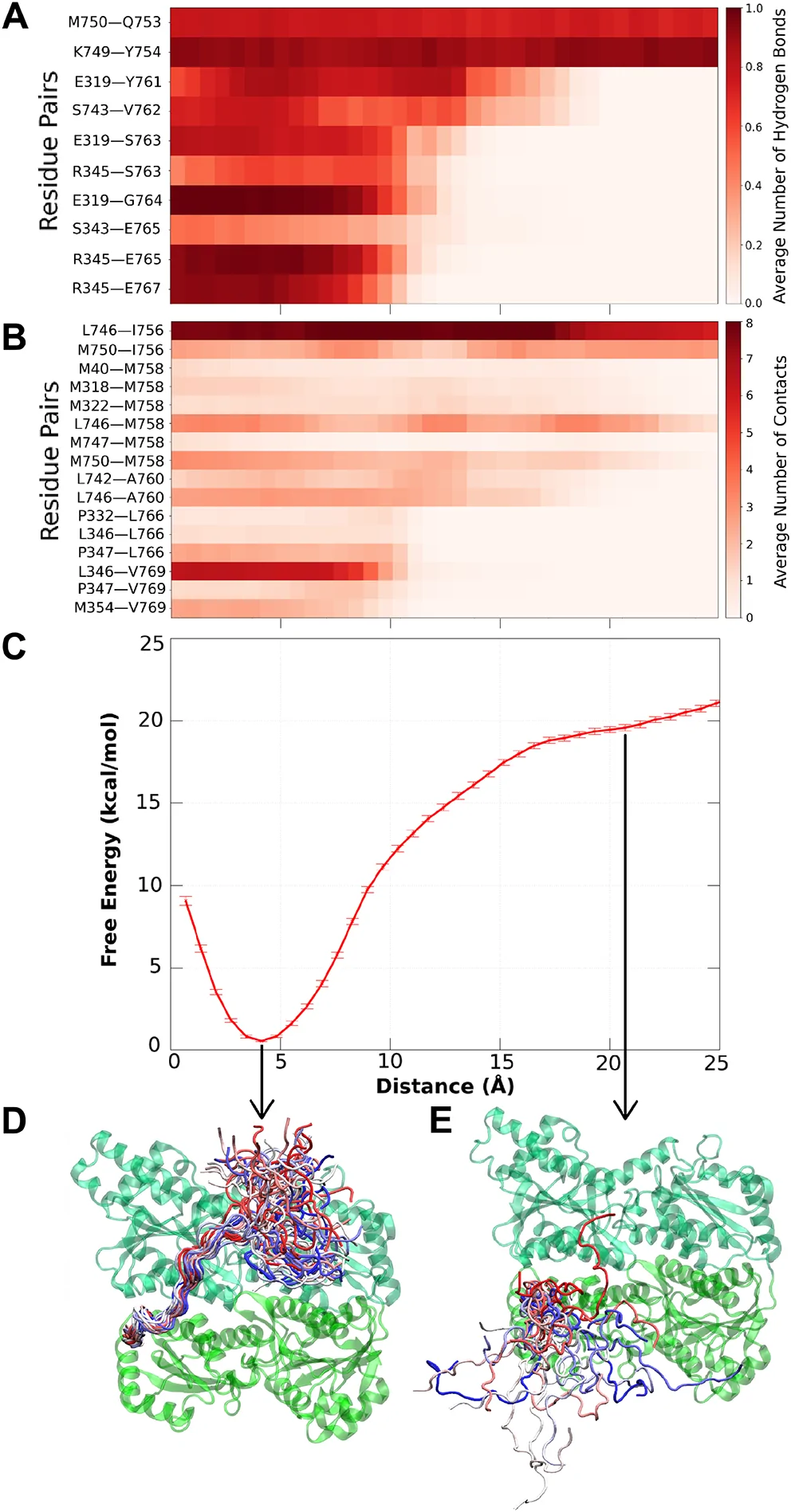
Results of REUS simulation of CTT detachment from the surface of the PFKL T-state. (A) and (B) Evolution of native interactions that stabilize CTT binding in the T-state as a function of a collective variable describing the displacement of the residue segment 753–771 from the enzyme surface. (C) Free-energy profile along the CTT displacement coordinate from the T-state structure. Conformational ensembles sampled by the CTT in (D) the bound state and (E) after unbinding.

To examine how the stabilizing interactions with the CTT are affected in the R-state, we performed simulations with CTT geometry transferred directly from the T-state cryoEM structure (PDB ID: 8W2H)^13^ onto the R-state cryoEM structure (PDB ID: 8W2I).^13^ For this system, labeled as R­(transposed), the interaction analysis shows that the native stabilizing interactions become more difficult to maintain due to incompatible geometries of CTT and the protein surface (Figure S12A,B). This is especially true for hydrogen bonds formed by residues E319–Y761, E319–G764, and R345–E765, the latter being involved in the β-strand motif observed in the T-state. The reduced compatibility results in a shallower minimum in the bound state, where the CTT interacts with the conserved surface region, compared to the T-state (Figure S12C,E). The shallower minimum is also present in the free-energy profile projected along the CTT native contact fraction (Figure S2).

Finally, we also performed a REUS simulation initiated from the R­(transition 2) geometry, where the CTT conformation and the protein surface coevolved during the transition and subsequent equilibration. In this case, the ability of the CTT and the protein surface to adjust during the transition may have reduced mismatches in their interfacial side-chain geometries and interactions. Although this simulation was initiated from a partially displaced CTT geometry with a disrupted β-strand motif, it still identifies a T-state-like CTT conformation and interaction pattern as the free-energy minimum. Only a few interactions, such as the hydrogen bond between residues E319–G764 in the intermediate region and the hydrogen bond between S743–V762 were modified due to the altered protein conformation (Figure S13A–E). Thus, R­(transition 2), whose domain geometry approaches but retains deviations from the experimental R-state, is best treated as an R-like mechanistic comparator: recovery of a T-state-like local docking pose at the biased-coordinate free-energy minimum helps identify the β-strand motif and other interactions important for CTT positioning but does not restore the interdomain bridge, because the rearranged domain geometry prevents simultaneous formation of the P347–L766 and K715–E767 interactions.

Furthermore, we modeled H768 as a neutral residue. However, depending on its protonation under physiological conditions, histidine can adopt a positively charged state. In its protonated state, H768 could form a salt bridge with E350, which is not included in our current model. In an additional 40 ns unbiased MD trajectory initiated from the T-state-bound conformation with positively charged H768, H768 formed a salt bridge with E350 (Figure S14). This observation identifies the H768–E350 interaction as a potential additional contributor to CTT stability.

Finally, the C-terminal end of the CTT remained flexible in our simulations, although we occasionally observed interactions between the final residue of the CTT, F780, and the residue K677 in the activating site (see Figure S15).

### Gaussian Accelerated Molecular Dynamics Sampling Confirms Expanded CTT Flexibility in the Absence of the β-Strand Motif

To more thoroughly explore the CTT conformations on the enzyme surface, we employed Gaussian accelerated molecular dynamics (GaMD), which accelerates conformational sampling without the need for a predefined reaction coordinate.^18,19^ We initiated the simulations from two distinct initial states: the T-state, in which the CTT forms the short β-strand motif, and R­(transposed), i.e., the R-state with the CTT transferred directly from the T-state cryoEM structure onto the R-state cryoEM structure. This transfer results in a disruption of the β-strand hydrogen bonding pattern (see the REUS results above). Throughout the GaMD simulations, the conformation of the protein surface adjacent to the CTT remained close to that of the corresponding T- or R-state cryoEM structure (Figure S16), consistent with the positional restraints described in the Methods.

The GaMD simulations, using three simulation replicas for each system, confirm that the CTT more readily escapes its initial binding pose and samples an expanded range of conformations when stabilizing interactions are perturbed by the T-to-R transition (see Figure S3). Specifically, the disruption of the hydrogen bonds formed by residues E319–G764 and R345–E767 in the R­(transposed) system correlates with a significantly broadened distribution of distances between the bridge-forming residues K715–E767 and hydrophobically interacting residues P347–L766 (see Figure S3A, B, D, and E). Moreover, in the T-state, the M354–V769 residue pair remains in proximity, whereas in R­(transposed), this residue pair exhibits significant distance fluctuations. These observations suggest that the M354–V769 contact contributes to stabilization of the T-state-like CTT conformation, while its disruption increases the flexibility of the CTT. In both states, the C-terminal residue F780 sampled a broad range of distances to residue K677 at the activating site (Figure S3C,F). Figures S4 and S5 show the time evolution of the distance between the aforementioned residue pairs in the T-state and R­(transposed), respectively, during 2 μs of biased simulations for the three independent replicas. Overall, because the REUS and GaMD simulations used CHARMM36m and AMBER ff14SB, respectively, their agreement should be interpreted qualitatively; it supports the key role of the identified stabilizing interactions but does not establish quantitatively equivalent contact stabilities or conformational ensembles.

### Specific Interactions Are Required for CTT Binding Stability

To determine whether the CTT remains stably bound on the enzyme surface even in coarse-grained simulations, where specific interactions such as hydrogen bonds are described only implicitly using orientation-averaged potentials, we performed simulations using the coarse-grained MARTINI 3 force field.^20^ These simulations showed reduced stability of the bound CTT for both the T-state and R­(transition 2) systems. Specifically, detachment of the CTT from the enzyme surface was observed in all simulation replicas. However, in the T-state, detachment occurred more slowly than in the R-state.

This weak stability of CTT binding was observed with both the standard version of MARTINI 3 ^20^ and a more recent version with rescaled protein–protein interactions^51^ to better capture the behavior of intrinsically disordered proteins. Figure S17A shows the average CTT contact probability per residue over three replicas for the T and R­(transition 2) enzyme conformations using the standard MARTINI 3 model, while Figures S17B–G and S18A–F show representative snapshots from the standard and rescaled models, respectively. The snapshots were taken from 25 μs simulations at intervals of 2.5 μs. Upon detachment from the bound state, the CTT sampled a broad conformational ensemble, including fully extended conformations in solution, detached and self-interacting conformations, and partially bound states in which the CTT contacts one of the domains.

After introducing specific interaction terms^55^ to describe the hydrogen bonds between the CTT β-strand residues and the enzyme surface, the stability of CTT binding increased significantly in this modified MARTINI 3 model, and the CTT retained its T-state-like conformation on the enzyme surface (see Figures 6A,B and S19). Because these native hydrogen-bond interactions are explicitly introduced by OLIVES, this comparison tests whether their inclusion improves retention of the T-state-like CTT pose rather than demonstrating that they are uniquely responsible for CTT stabilization. Within this limitation, the result is nevertheless consistent with a key contribution of the β-strand motif and other specific interactions in stabilizing CTT binding.

**Figure 6:**
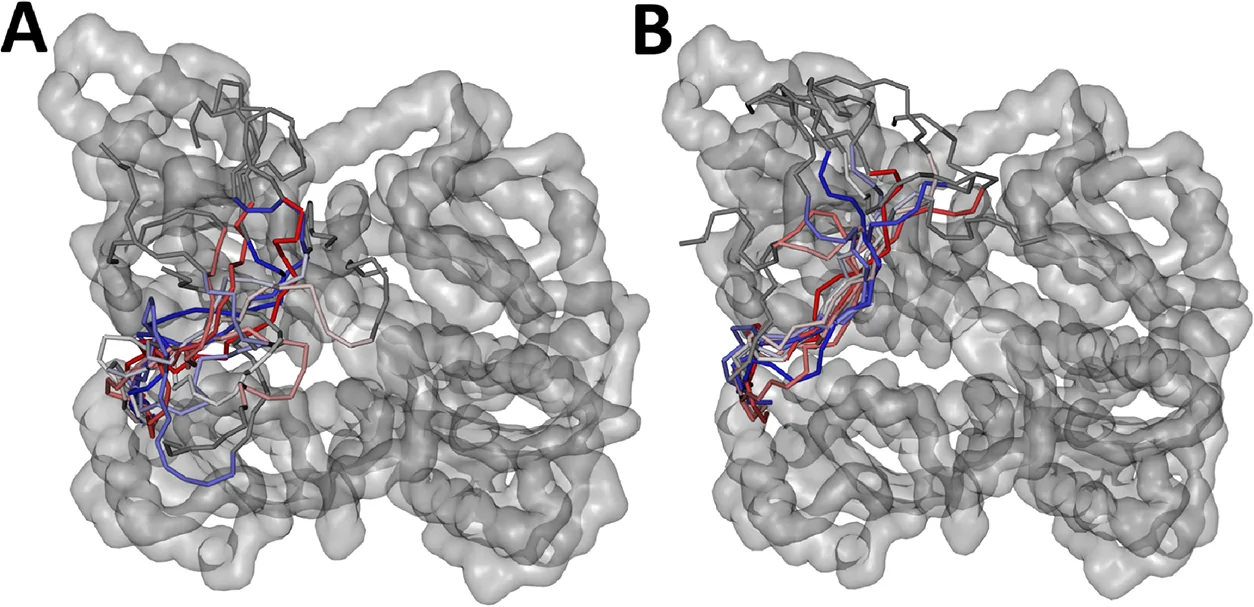
Snapshots of the CTT in the T-state using (**A**) the MARTINI 3 force field^20^ and (**B**) the MARTINI 3 force field with extra hydrogen-bonding terms obtained from OLIVES.^55^ Snapshots were extracted from 15 μs simulations at intervals of 1 μs, with the first 5 μs of each trajectory discarded as equilibration.

## Conclusions

In this work, we investigated how the disordered C-terminal tail (CTT) of human PFK1 achieves the spatial precision required for allosteric regulation. By combining enhanced-sampling molecular dynamics simulations, conservation-guided spatial analysis, allosteric network analysis, and coarse-grained modeling, we show that functional CTT positioning is encoded by a small set of residue-specific hydrogen-bonding and hydrophobic interactions that stabilize a defined surface-bound arrangement in the inhibited T-state.

This positioning allows two conserved CTT residues, L766 and E767, to contribute to ATP-mediated inhibition, consistent with previous biochemical evidence.^15^ L766 dynamically couples the CTT to a conserved surface region adjacent to the active site, providing a potential route by which CTT binding can influence active-site conformation. E767 forms a salt bridge with K715 in the regulatory domain, completing an interdomain bridge characteristic of the T-state. These effects depend on the precise placement of the CTT on the enzyme surface, which is maintained by specific interactions, including a short β-strand motif formed by residues 765–767 and hydrogen bonds with the conserved surface residue E319.

Because the CTT docking site is entirely intrasubunit and the applied protein restraints maintain its local tetramer-derived geometry, the apo monomers provide suitable reduced proxies for identifying the direct structural determinants of CTT docking. Nevertheless, these simulations do not directly quantify how ligand occupancy, intersubunit packing, or tetramer-scale conformational relaxation modulates these contacts.

Within the standard and rescaled MARTINI 3 models tested here, coarse-grained simulations lacking explicit representation of these interactions lead to greater CTT flexibility and unbinding, indicating that nonspecific surface attraction is insufficient to maintain the allosterically competent tail position. Thus, the PFK1 CTT acts as a regulatory element whose functional position on the enzyme surface is defined by specific residue-level contacts. Taken together with previous structural and biochemical evidence, our results identify PFK1 as an example in which sequence-specific surface docking of a disordered terminal segment provides a physicochemical mechanism for enzyme allostery. This perspective has implications for interpreting CTT truncations, post-translational modifications, and isoform-specific regulation, all of which may alter the functional surface-bound arrangement of the tail. It also suggests that fluorescent-protein fusions may alter this arrangement, a possibility that should be evaluated in future work.

## Supplementary Material



## Notes

Convergence analysis and histograms of free energy profiles (Figure S1); native contact map between the CTT and the protein and PMF profile projected onto the fraction of native contacts (Figure S2); GaMD 2D histograms of distance distributions for T and R­(transposed) (Figure S3); time evolution of the distance between different residue pairs in the T-state (Figure S4); time evolution of the distance between different residue pairs in the R­(transposed) (Figure S5); native contacts gained and lost during the T-R transition and suboptimal paths (Figure S6); structural and RMSD analysis of regions involved in CTT docking and conformational changes (Figure S7); RMSD for R­(transition 1) and R­(transition 2) during the transition, and 2 μs of equilibration (Figure S8); domain angle, transition coordinate, and RMSD evolution during the T-to-R transition and equilibration (Figure S9); evolution of selected residue contacts during the T-to-R transition (Figure S10); histograms of distances between prolines P332 and P347 (Figure S11); results of REUS simulation of CTT detachment from the surface of PFKL R­(transposed) (Figure S12); results of REUS simulation of CTT detachment from the surface of PFKL R­(transition 2) (Figure S13); salt-bridge analysis involving E350 and H768 (Figure S14); minimum distance between residues K677 and F780 across conformational states (Figure S15); RMSD analysis of the E319-E350 region in GaMD simulations (Figure S16); average CTT contact probability per residue and snapshots for T and R­(transition 2) with standard MARTINI 3 (Figure S17); snapshots for T and R­(transition 2) with the recent version of MARTINI 3 with protein–protein interactions rescaled (Figure S18); CTT RMSD analysis using standard MARTINI 3, scaled MARTINI 3, and OLIVES models (Figure S19); details of the equilibration protocol (Table S1); average distances between atoms forming hydrogen bonds HB1, HB2, and HB3 in REUS windows corresponding to free-energy minima (Table S2); and average occupancies of hydrogen bonds HB1, HB2, and HB3 in REUS windows corresponding to free-energy minima (Table S3) (PDF)
