# Supplementary material for: Poised for Allostery: Specific Interactions Underlie Precise Positioning of the C‑Terminal Tail in Phosphofructokinase‑1
Source: J Phys Chem B. 2026 Oct 26;130(44):11681–92. doi: 10.1021/acs.jpcb.6c03671 (PMC13746788; doi:10.1021/acs.jpcb.6c03671)
Supplement: Supplementary file 1 [file jp6c03671_si_001.pdf]

Supporting Information to: Poised for Allostery: Specific Interactions Underlie Precise Positioning of the C-Terminal Tail in Phosphofructokinase-1

Mehrnoosh Khodam Hazrati,[†] Truong-An Nguyen,[†,‡] and Stepan Timr[*,†]

†*J. Heyrovsky Institute of Physical Chemistry, Czech Academy of Sciences, Dolejskova 2155/3, 182 00 Prague 8, Czech Republic*

‡*Faculty of Science, Charles University, Albertov 6, 128 00 Prague 2, Czech Republic*

E-mail: stepan.timr@jh-inst.cas.cz

The average exchange probabilities in the REUS for the 64 windows upon CTT displacement in the T-state, R(transposed) and R(transition 2) are listed as follows:

T-state:

.49 .49 .48 .48 .49 .48 .47 .48 .47 .40 .47 .47 .45 .43 .39 .42 .29 .30 .32 .25 .44 .42 .42 .40 .40 .41 .42 .42 .28 .43 .43 .30 .32 .32 .33 .33 .32 .43 .44 .43 .33 .34 .34 .34 .35 .35 .35 .35 .34 .34 .33 .33 .33 .44 .44 .33 .33 .34 .34 .34 .34 .34 .35

R(transposed):

.46 .46 .49 .48 .49 .48 .37 .39 .43 .42 .40 .48 .48 .34 .45 .44 .42 .28 .44 .43 .42 .42 .42 .42 .42 .42 .31 .33 .44 .43 .42 .43 .32 .33 .43 .43 .43 .43 .43 .43 .44 .44 .35 .36 .36 .36 .37 .36 .35 .44 .44 .35 .36 .36 .36 .36 .45 .44 .34 .45 .44 .44 .45

R(transition 2):

.49 .50 .49 .49 .46 .49 .48 .49 .48 .48 .48 .48 .46 .46 .43 .32 .41 .43 .43 .43 .43 .44 .43 .44 .44 .44 .32 .43 .43 .43 .43 .43 .43 .43 .43 .43 .44 .44 .44 .44 .44 .35 .44 .44 .36 .36 .44 .44 .36 .36 .44 .44 .44 .44 .45 .44 .44 .44 .44 .45 .45 .45 .46

Supporting Figures

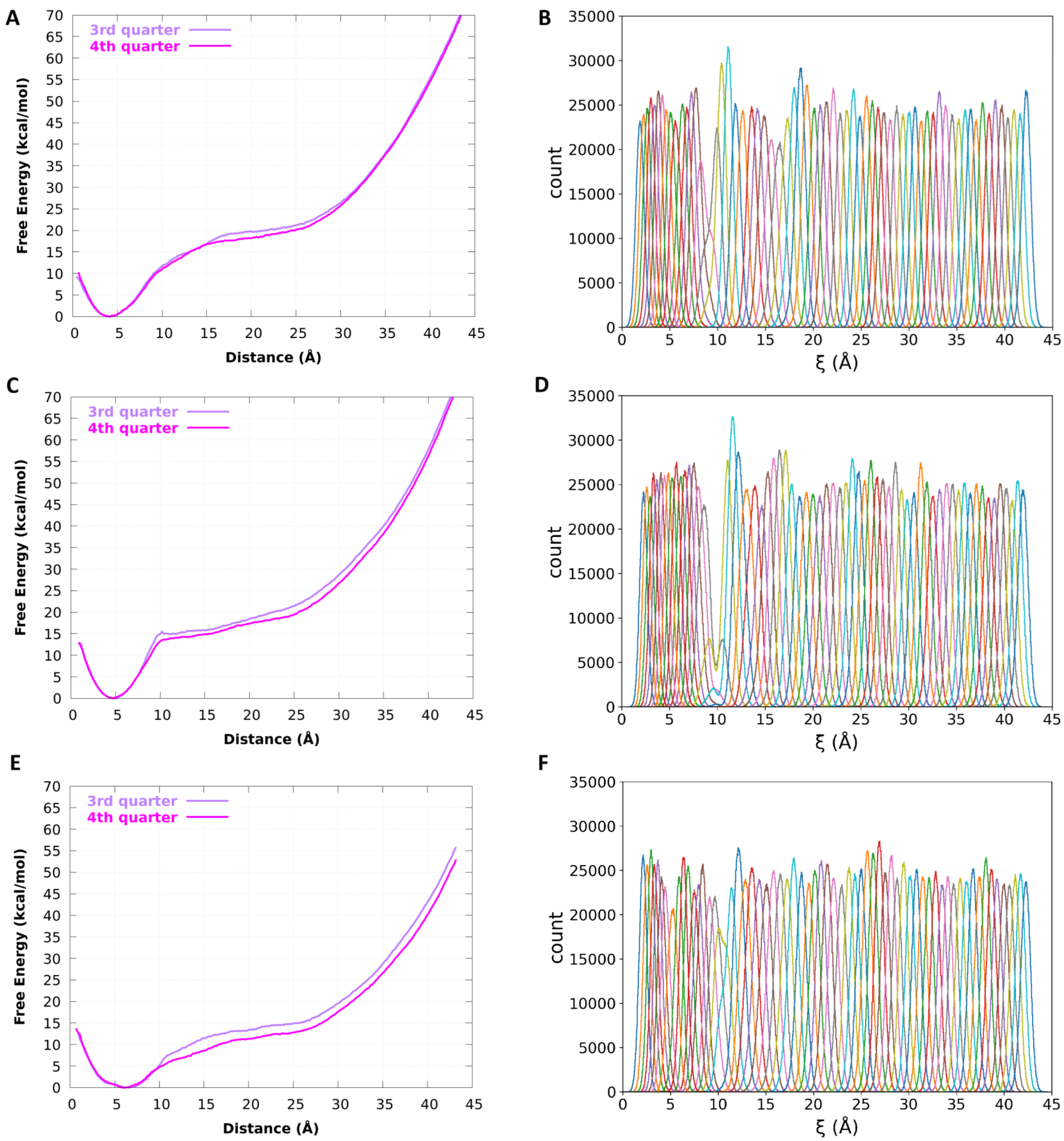

Figure S1: Free energy profiles obtained from the third and fourth quarters of the trajectories indicating convergence, and histograms obtained from the second halves of the trajectories indicate proper overlap between windows for (**A** and **B**) T, (**C** and **D**) R(transition 2), and (**E** and **F**) R(transposed).

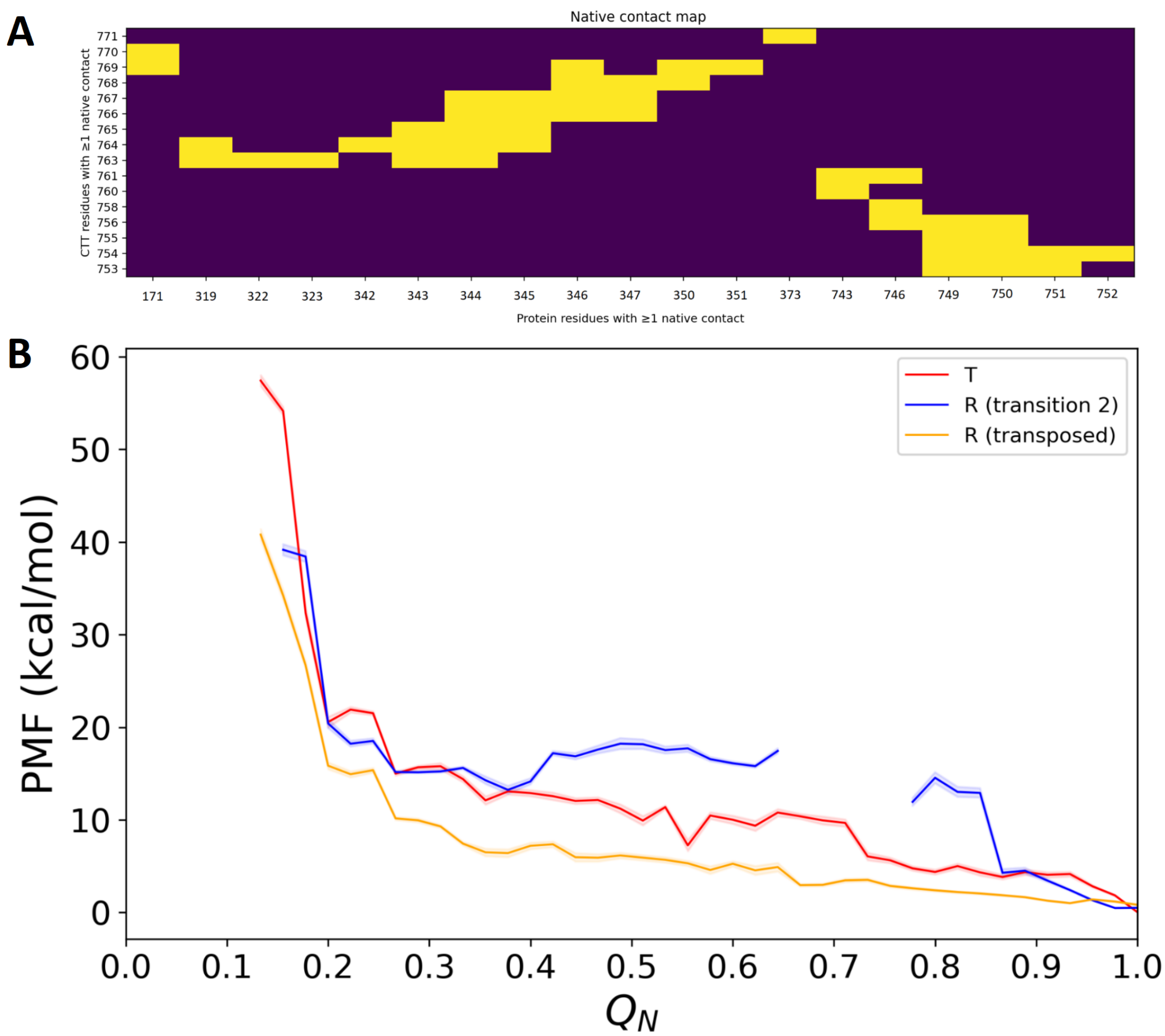

Figure S2: (**A**) Native contact map between the CTT (residues 753–771) and the remainder of the protein, based on the T-state cryoEM structure. (**B**) Potential of mean force (PMF) profile projected onto the fraction of native contacts, Q_{N}, obtained by reweighting the conformations sampled in the REUS simulation.

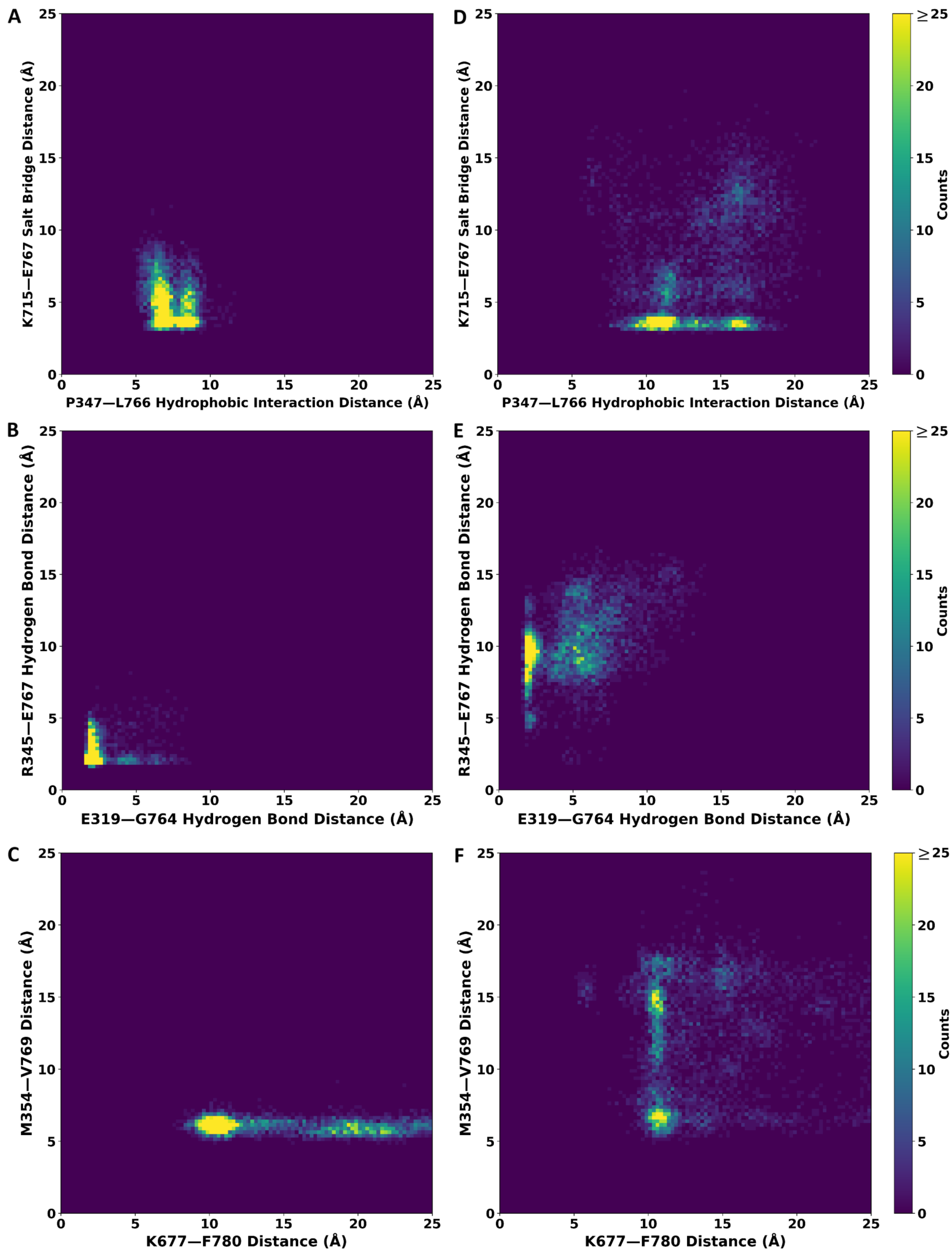

Figure S3: 2D histograms of distance distributions calculated from concatenated trajectories of three independent 2 μs replicas, with frames saved every 1 ns (6000 frames in total). The distributions indicate that disruption of stabilizing interactions increases CTT flexibility on the enzyme surface in the R-state. (**A**)–(**C**) T-state; (**D**)–(**F**) R(transposed). The hydrogen bond between R345 and E767 is referred to as HB1, and the salt bridge between K715 and E767 is referred to as SB1.

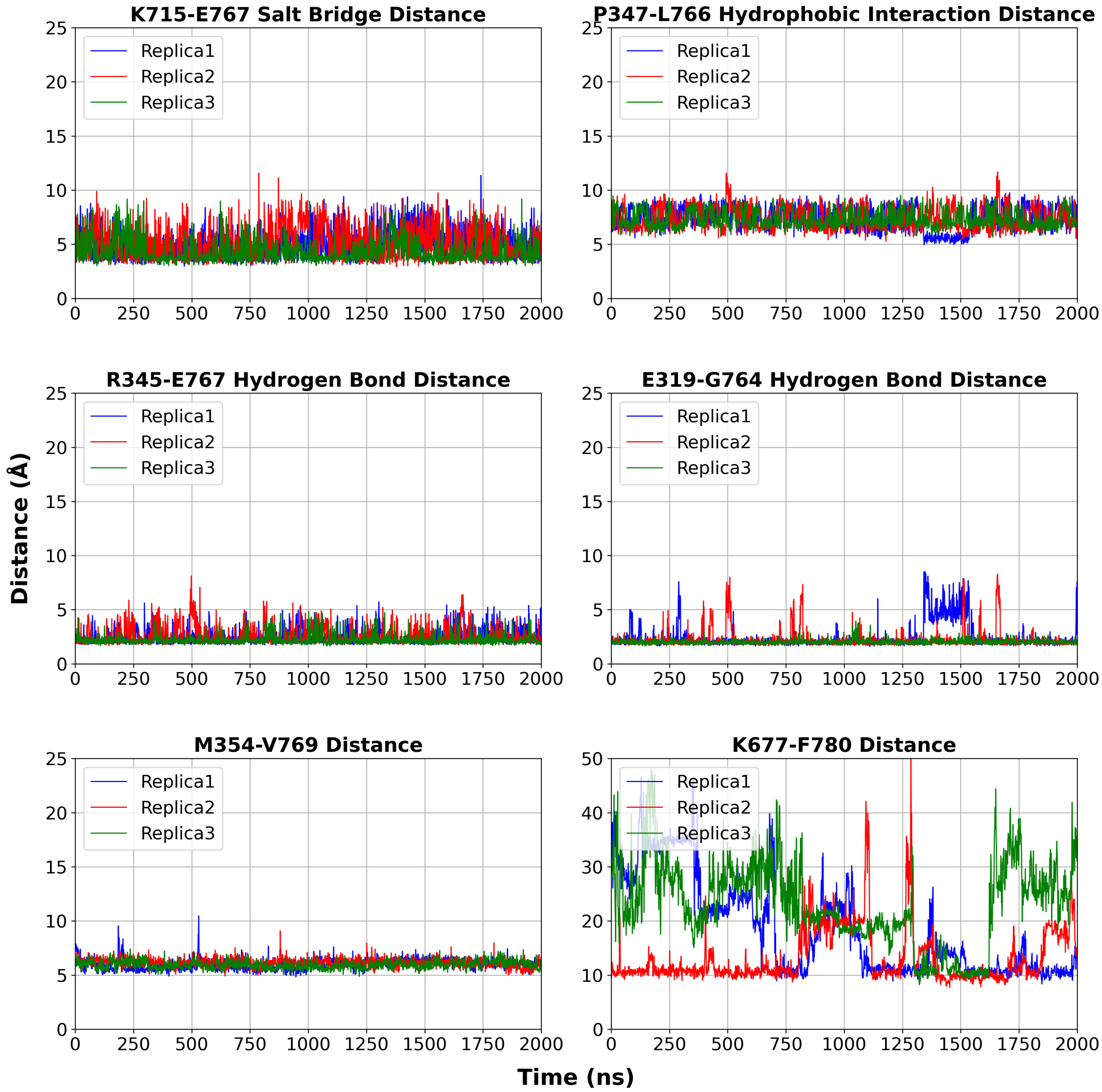

Figure S4: Time evolution of the distance between different residue pairs in the T-state during 2 μs of PPI-GaMD simulations for three independent replicas.

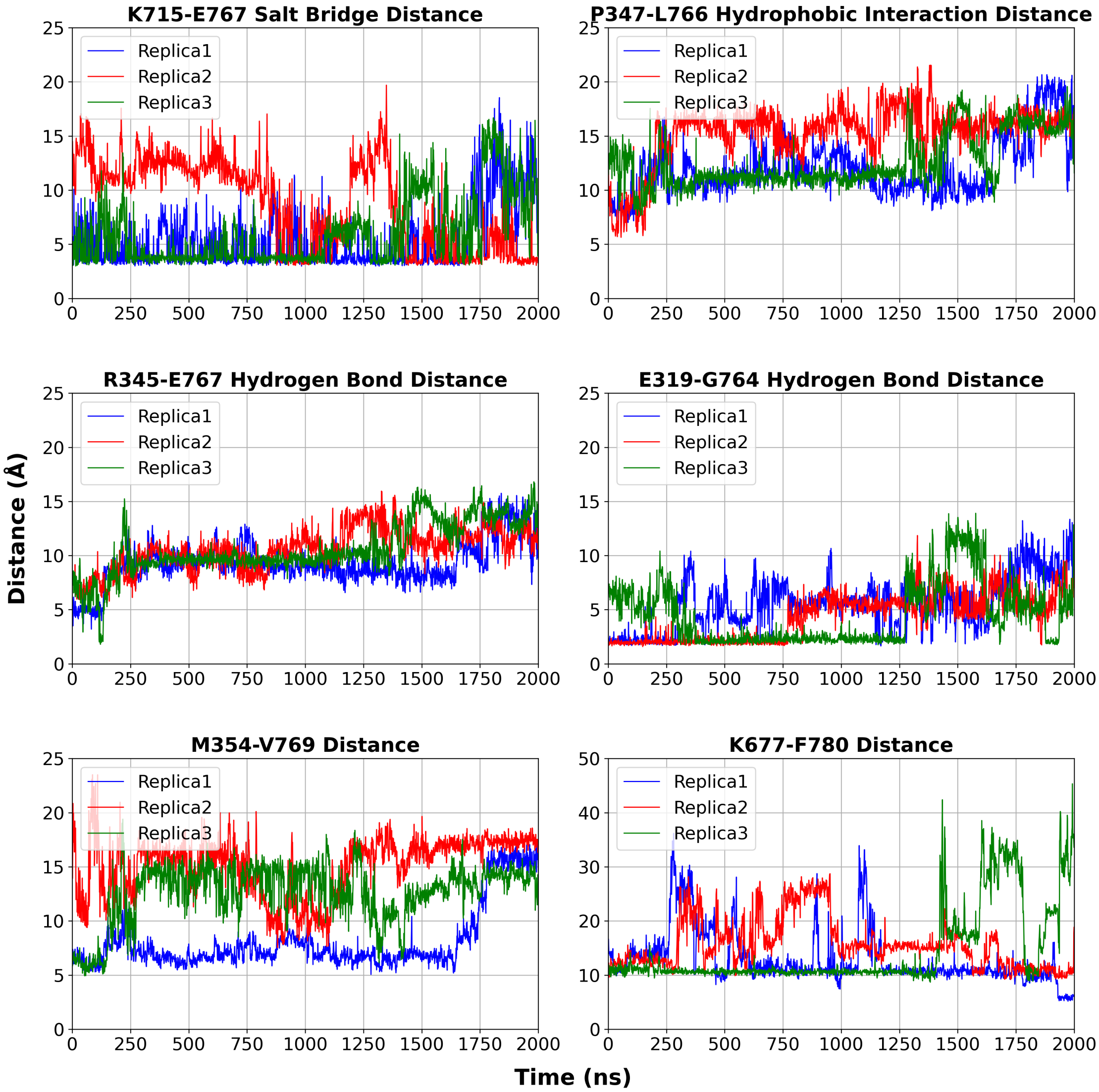

Figure S5: Time evolution of the distance between different residue pairs in the R(transposed) during 2 μs of PPI-GaMD simulations for three independent replicas.

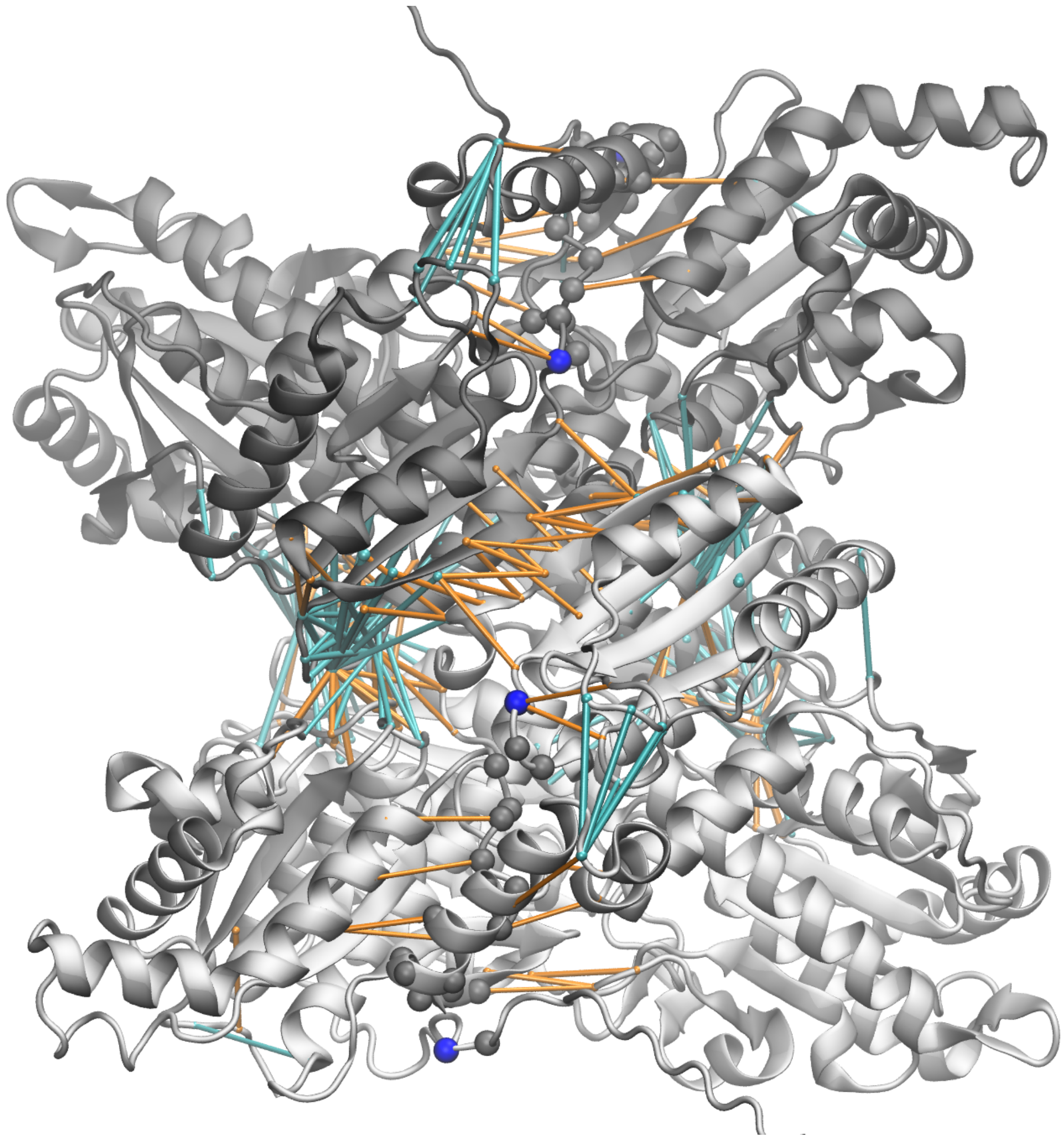

Figure S6: PFKL dimer in the T-state with colored lines showing native contacts gained (cyan) and lost (orange) upon a T-R transition. Gray beads highlight residues crossed by suboptimal paths connecting residues LEU766 and ARG210 (see Figure 2B in the main text).

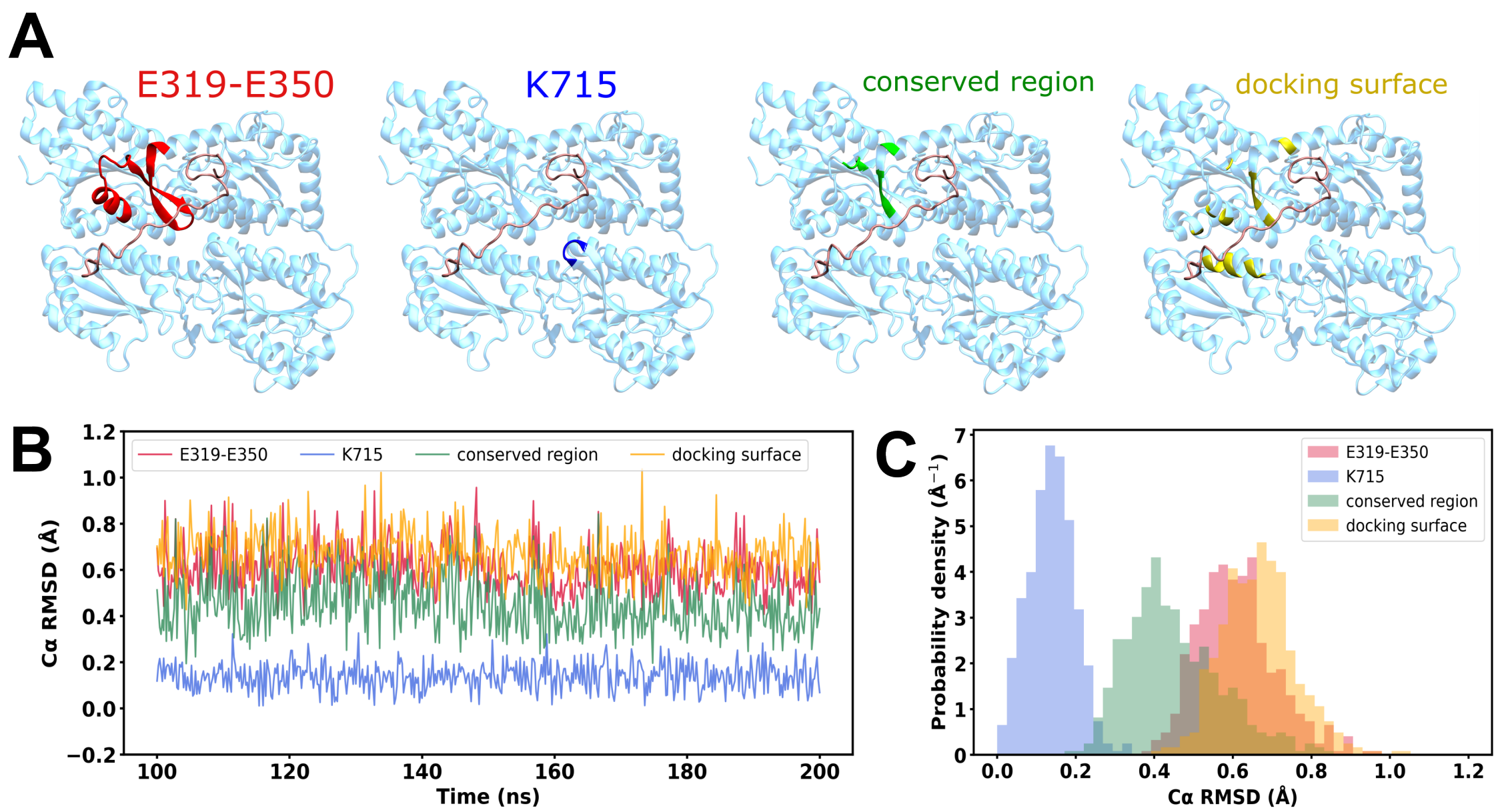

Figure S7: (**A**) Monomer structure highlighting the E319–E350 region (red), the K715 region (blue; residues 714–716), the conserved region adjacent to the active site (green; residues 331, 332, 334, 344–347, and 350), and the CTT-docking surface (yellow; residues 40, 318, 319, 322, 332, 343, 345–347, 354, 742, 743, 746, 747, 749, and 750). The CTT is shown in pink. (**B**) Root-mean-square displacement (RMSD; Å) of the Cα atoms in the highlighted regions relative to the T-state cryoEM structure over the 200-ns trajectory from the minimum-free-energy window of the T-state REUS simulation. (**C**) Probability distributions of the RMSD values for the corresponding regions.

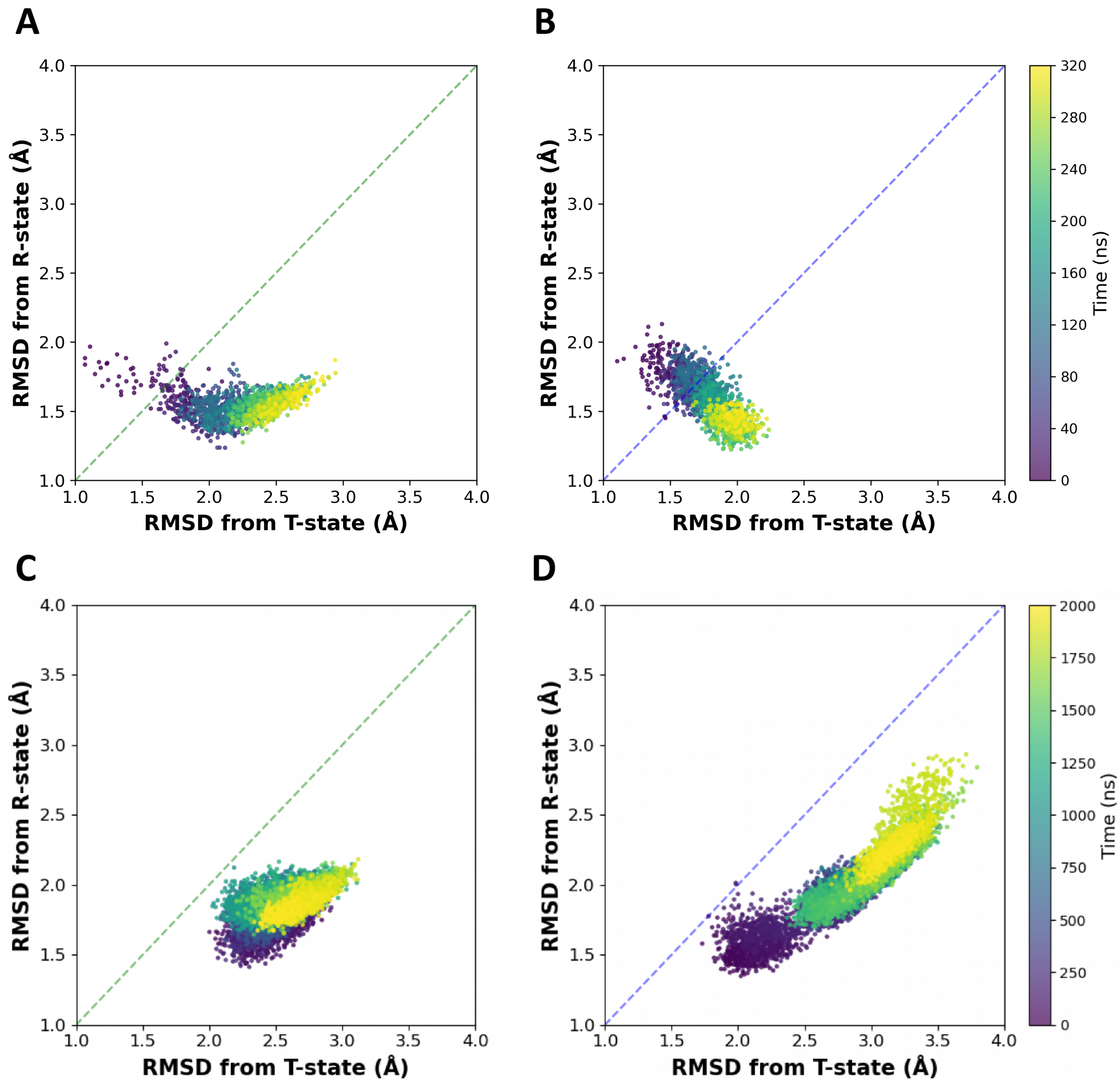

Figure S8: The RMSD during 320 ns of transition resulted in **(A)** R(transition 1), and **(B)** R(transition 2). The RMSD for **(C)** R(transition 1), and **(D)** R(transition 2) during 2 μs of equilibration.

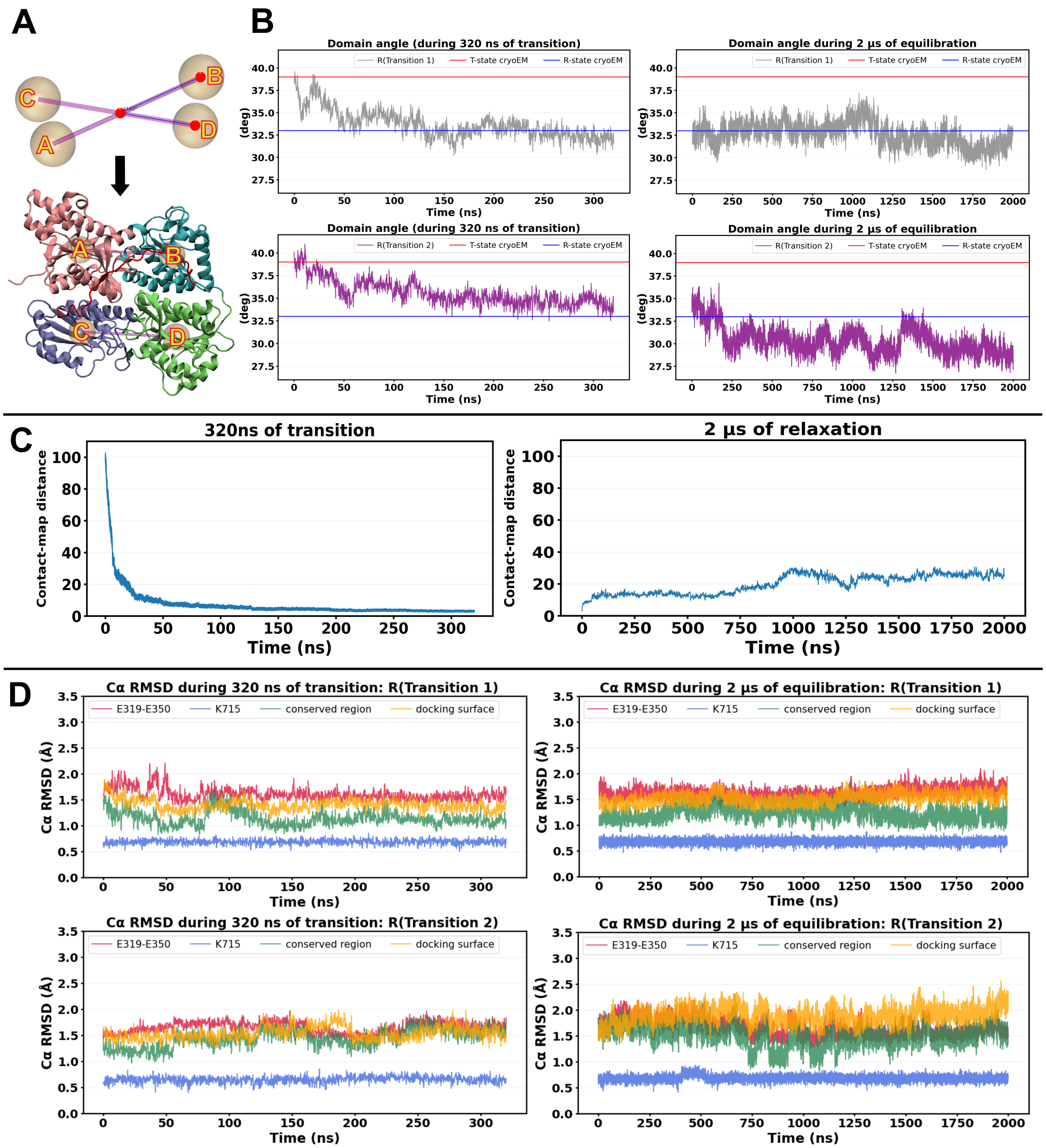

Figure S9: (**A**) Definition of the domain angle of a PFKL subunit using the centers of mass of four subdomains (positions A–D) to characterize the relative motion between the regulatory and catalytic domains. Subdomains A, B, C, and D comprise residues 13–178 and 302–354; 179–301 and 355–395; 396–540 and 666–729; and 541–665 and 730–753, respectively. (**B**) Evolution of the domain angle during the 320-ns T-to-R transition and subsequent 2-μs equilibration. The red and blue horizontal lines indicate the corresponding values in the T-state and R-state cryoEM structures, respectively. (**C**) Evolution of the contact-map distance used to drive the T-to-R transition during the 320-ns transition and subsequent 2-μs equilibration. (**D**) Evolution of the Cα RMSD of the CTT-adjacent regions relative to their conformations in the R-state cryoEM structure.

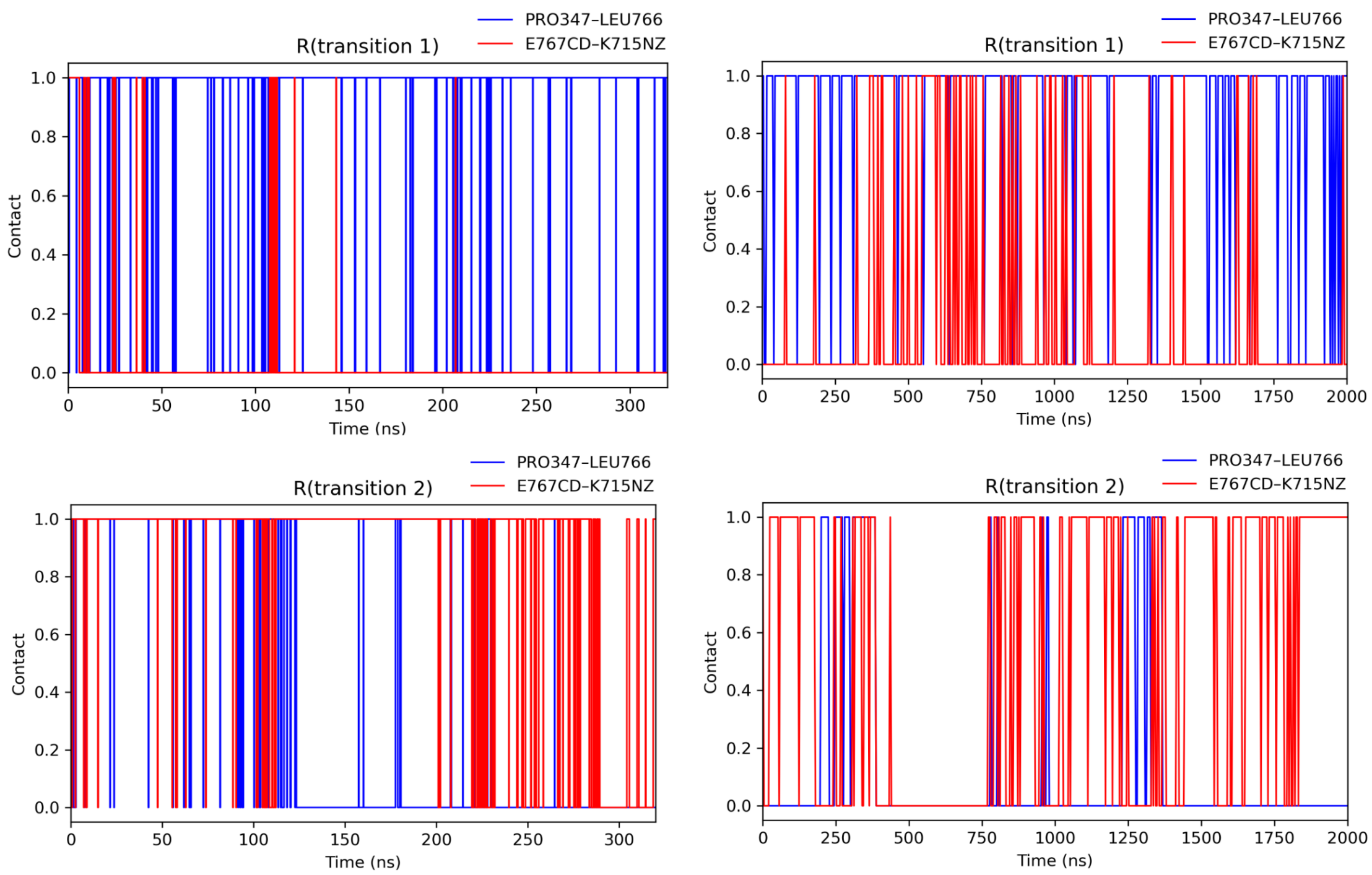

Figure S10: Evolution of the PRO347–LEU766 carbon atoms (blue) and E767:CD–K715:NZ (red) contacts during the 320-ns T-to-R transition and subsequent 2-μs relaxation.

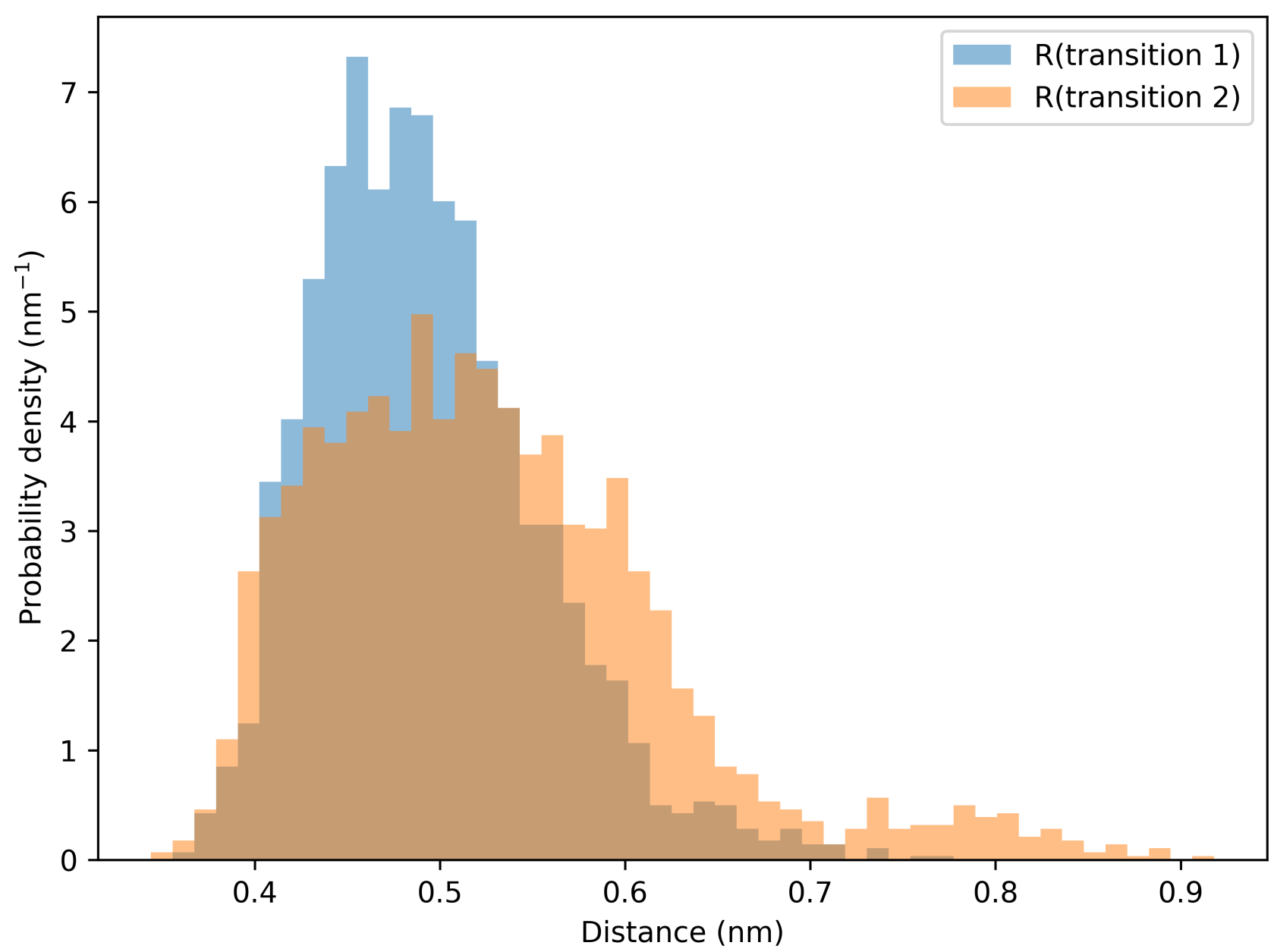

Figure S11: Histograms of distances between the C_β atoms of proline residues P332 and P347 in the two subunits of the PFKL dimer during a 2 μs relaxation trajectory following an induced T-to-R transition.

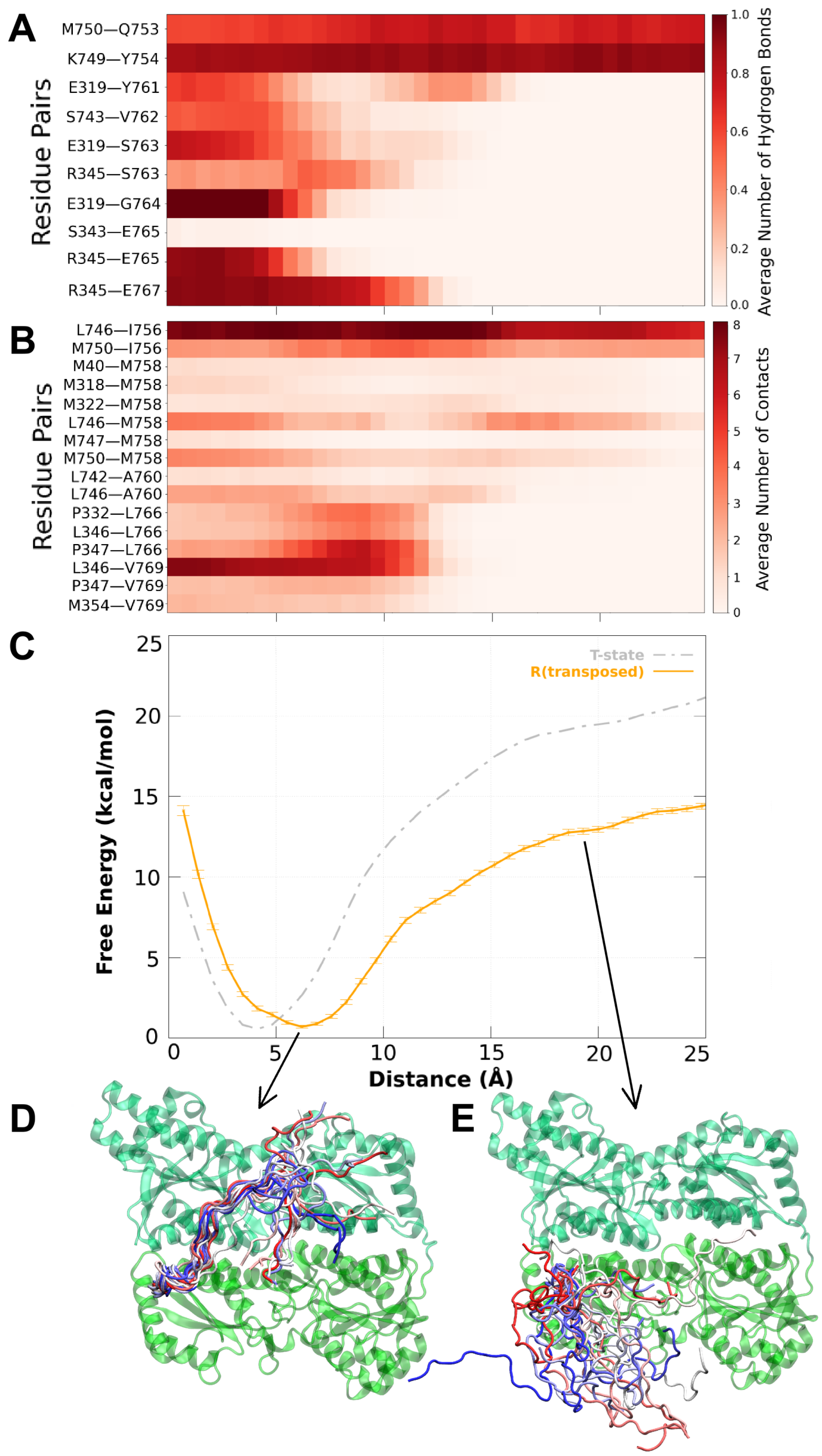

Figure S12: Results of REUS simulation of CTT detachment from the surface of PFKL R(transposed). **(A)** and **(B)** Evolution of native interactions in the R(transposed) as a function of a collective variable representing the displacement of the residue segment 753–771 from the enzyme surface. **(C)** Free energy profile along the CTT displacement from the R(transposed) structure. Conformational ensembles sampled by CTT in **(D)** the bound state and **(E)** after unbinding.

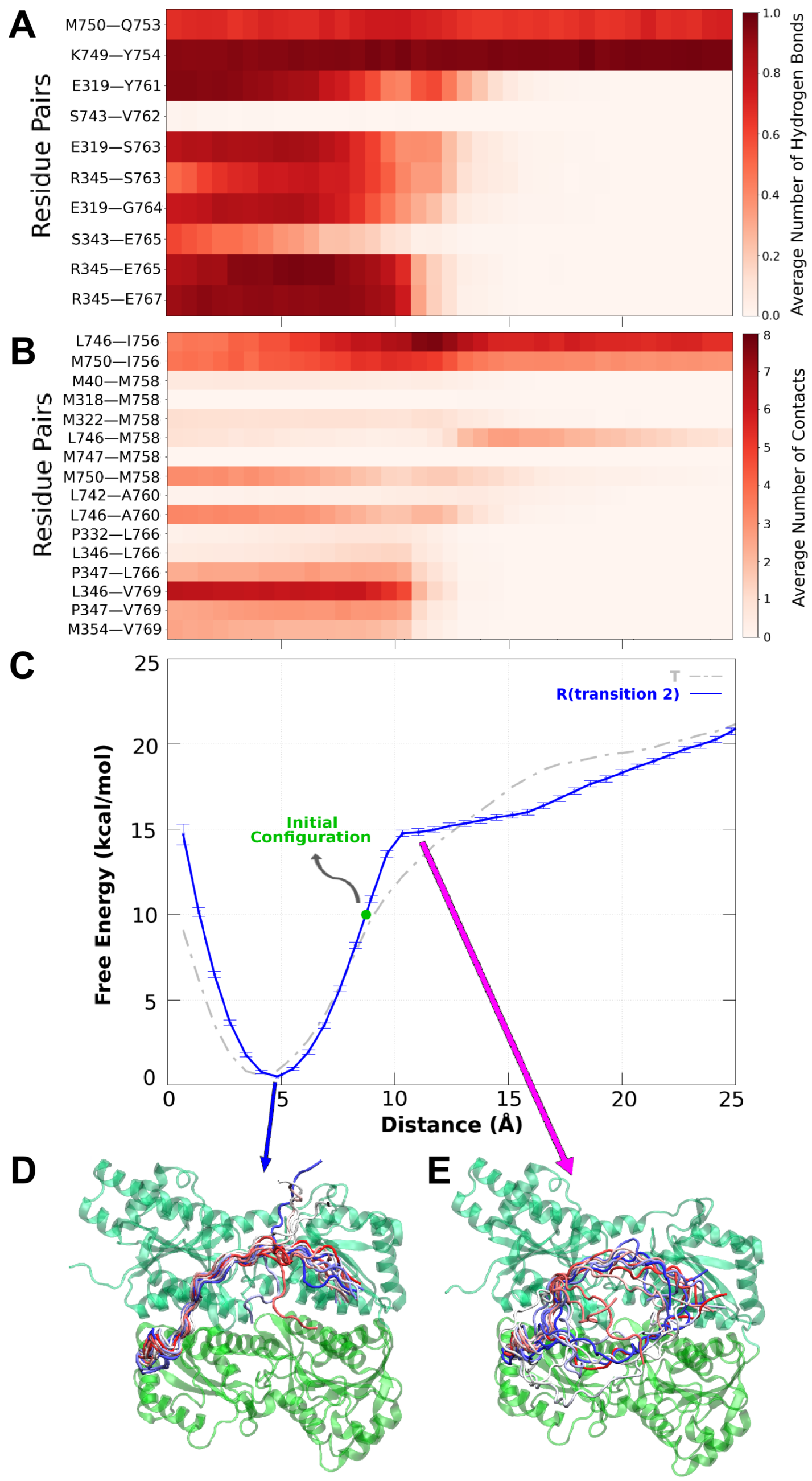

Figure S13: Results of REUS simulation of CTT detachment from the surface of PFKL R(transition 2). **(A)** and **(B)** Evolution of native interactions in the R(transition 2) as a function of a collective variable representing the displacement of the residue segment 753–771 from the enzyme surface. **(C)** Free energy profile along the CTT displacement from the R(transition 2) structure. Conformational ensembles sampled by CTT in **(D)** the bound state and **(E)** after unbinding.

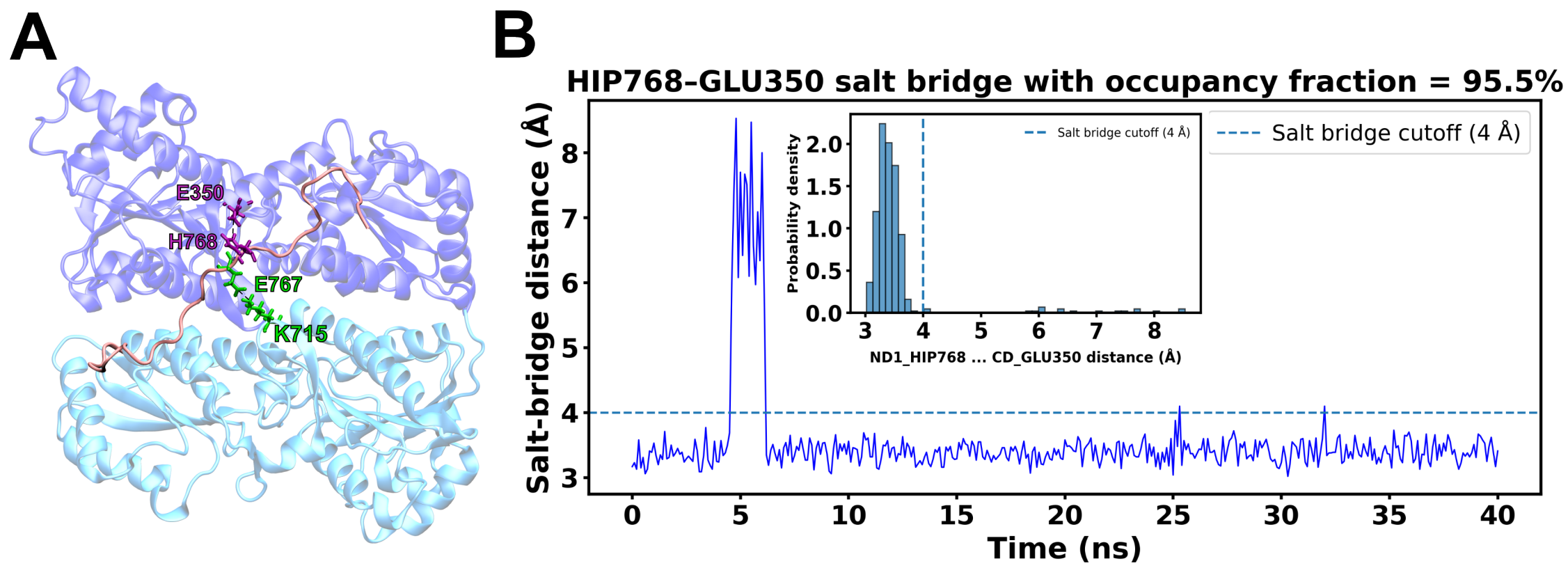

Figure S14: (**A**) Salt bridge between E350 and protonated H768 observed in an additional 40-ns unbiased MD simulation of the T-state structure with positively charged H768. (**B**) Time evolution of the salt-bridge distance between the E350 side-chain CD atom and the H768 side-chain ND1 atom during the 40-ns simulation. The inset shows a salt-bridge occupancy of 95% (inset).

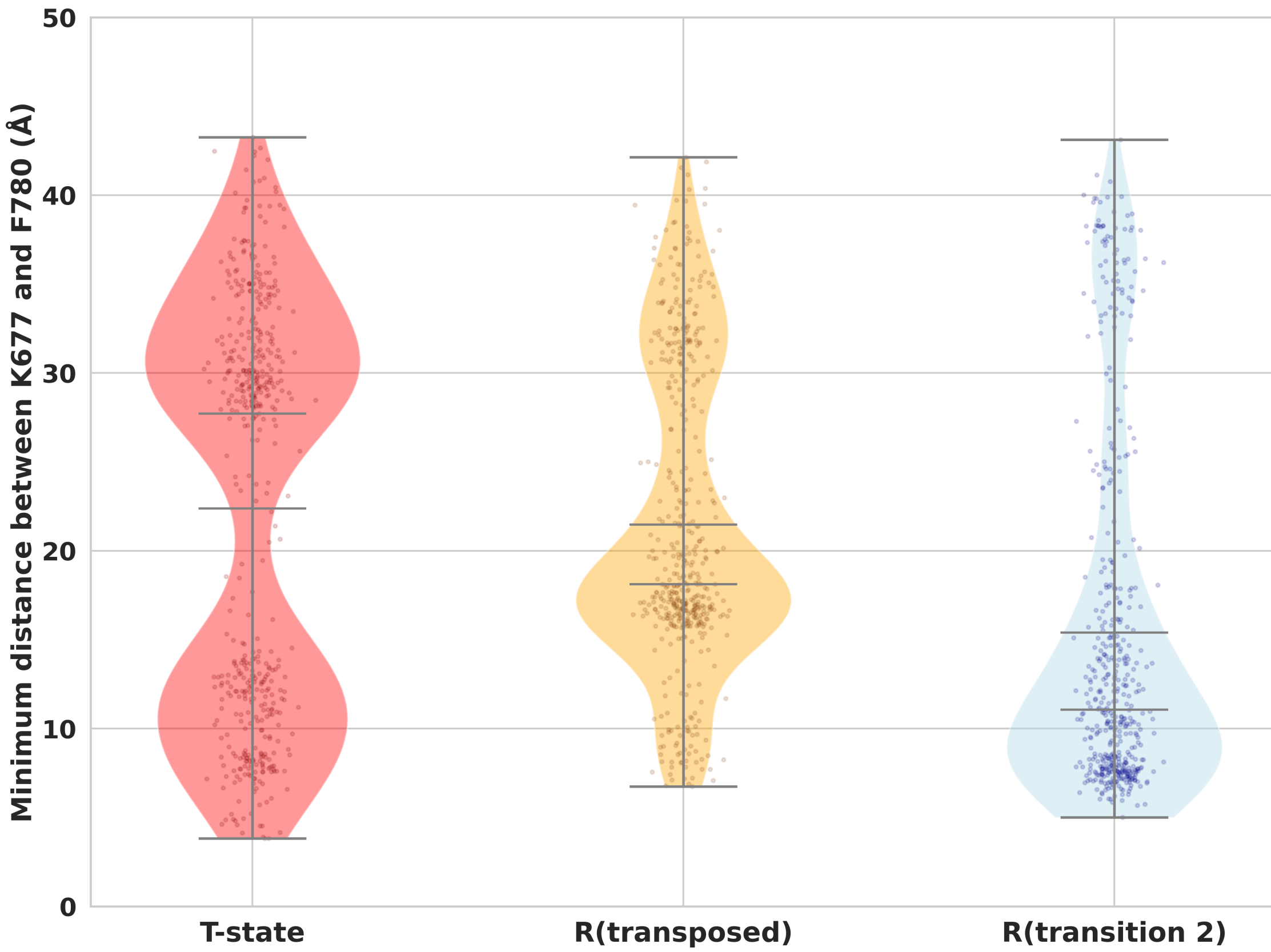

Figure S15: Minimum distance between residues K677 and F780 across conformational states. Violin plots show the distributions of minimum residue distances for the T-state, R(transposed), and R(transition 2). Individual data points are shown as dots; for each distribution, the gray vertical line spans the full data range (minimum to maximum), while the two internal horizontal bars indicate the mean and median.

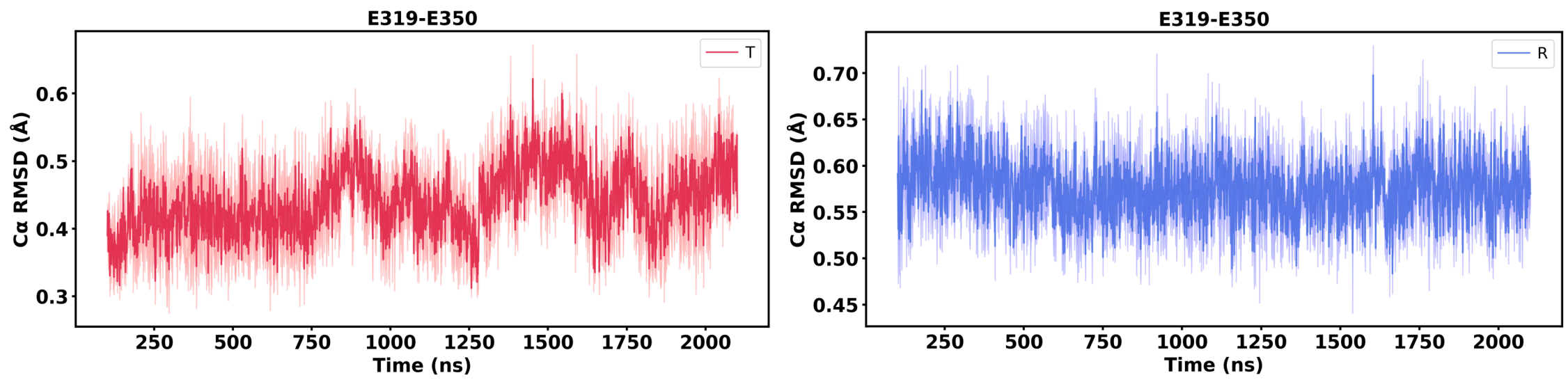

Figure S16: Root-mean-square displacement (RMSD; Å) of the backbone Cα atoms of residues E319–E350 relative to the T-state cryoEM structure (left) and the R-state cryoEM structure (right) as a function of time in the GaMD simulations.

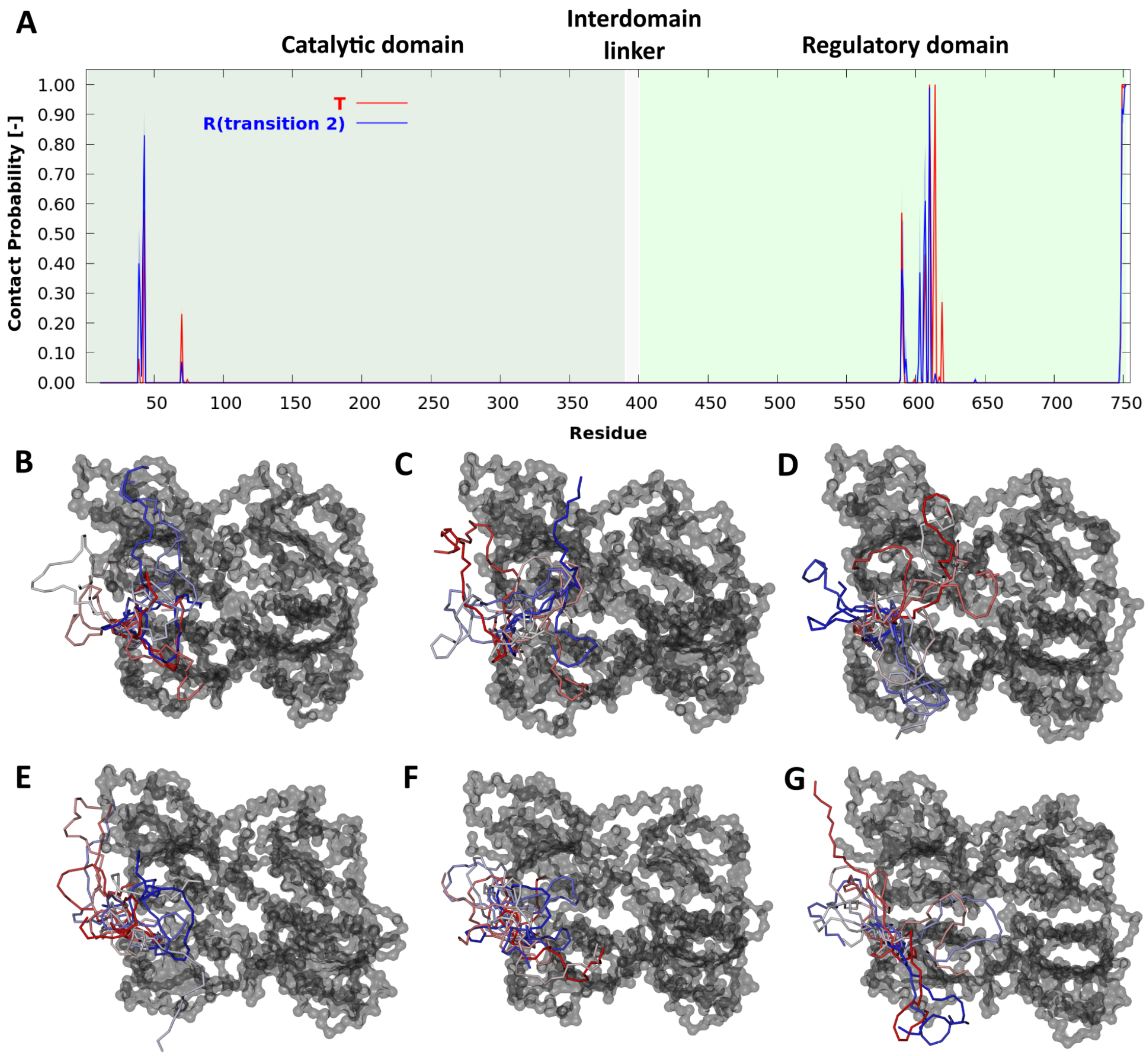

Figure S17: (**A**) Average CTT contact probability per residue over three replicas for the T and R(transition 2). Snapshots of the CTT in the T-state are shown for (**B**) replica 1, (**C**) replica 2, and (**D**) replica 3. Snapshots of the CTT in R(transition 2) are shown for (**E**) replica 1, (**F**) replica 2, and (**G**) replica 3. Snapshots were taken from 25 μs simulations at intervals of 2 μs using the standard MARTINI 3 force field. The first 5 μs of each trajectory were discarded as equilibration.

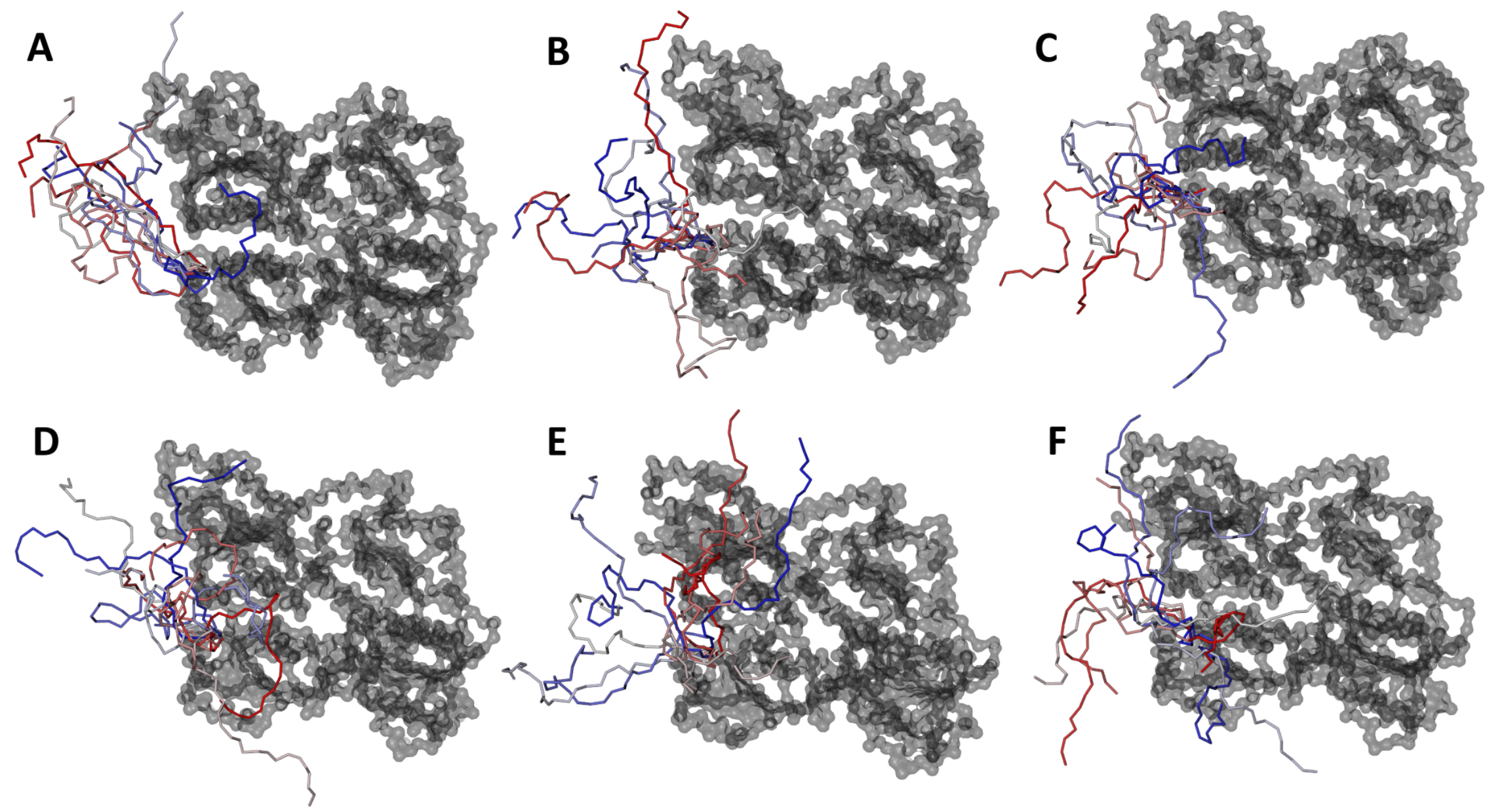

Figure S18: Snapshots of the CTT in the T-state are shown for (**A**) replica 1, (**B**) replica 2, and (**C**) replica 3. Snapshots of the CTT in R(transition 2) are shown for (**D**) replica 1, (**E**) replica 2, and (**F**) replica 3. Snapshots were taken from 25 μs simulations at intervals of 2 μs using the recent version of MARTINI 3 force field with protein–protein interactions rescaled. The first 5 μs of each trajectory were discarded as equilibration.

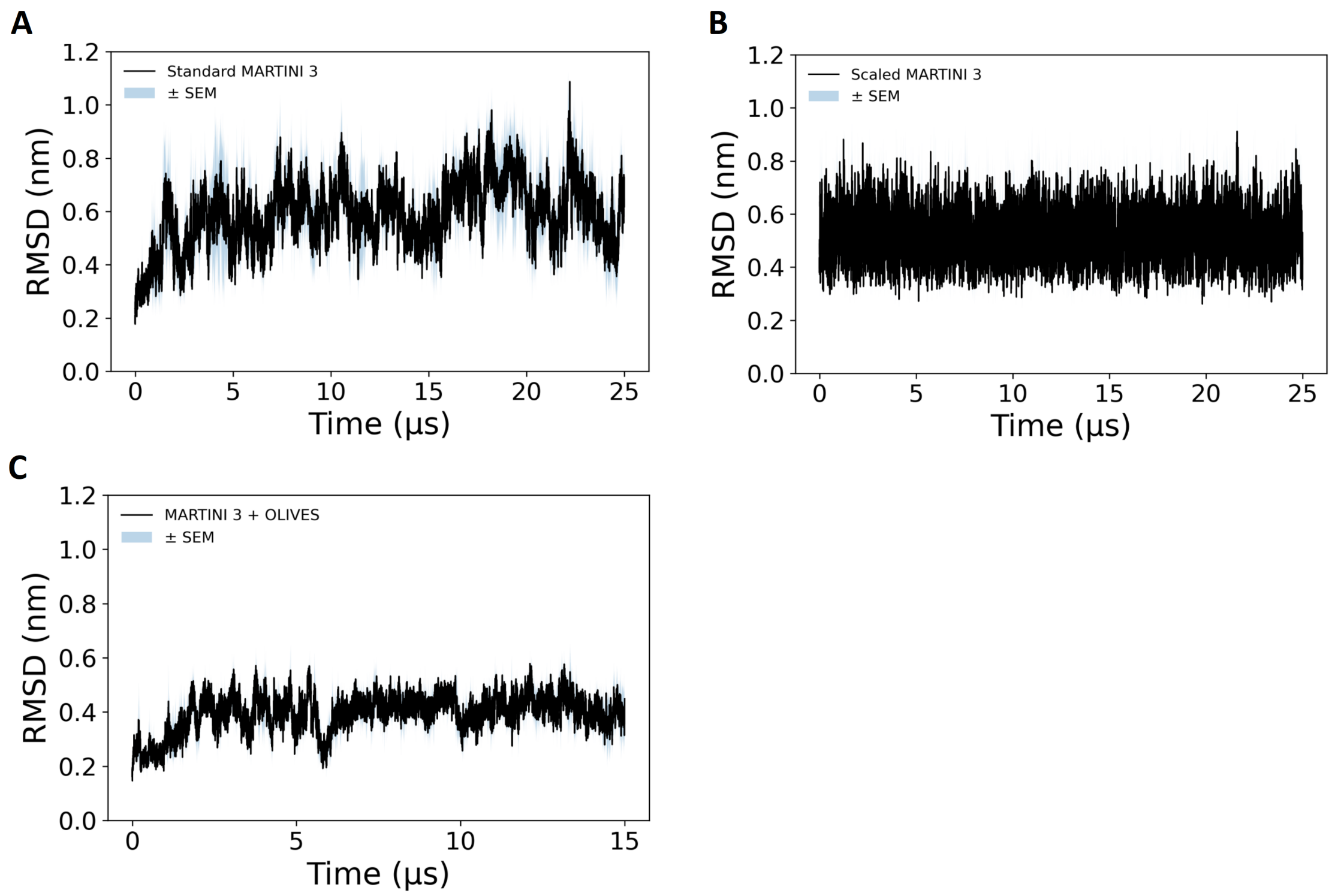

Figure S19: RMSD of the CTT backbone beads (Q753–R771) relative to their cryoEM T-state structure, calculated from three independent coarse-grained simulation replicas for: (**A**) standard MARTINI 3 model, (**B**) scaled MARTINI 3 model, and (**C**) OLIVES model.

Supporting Tables

Table S1: Overview of the equilibration protocol. k_{BB} – force constant of harmonic restraint potentials applied to the heavy atoms of the backbone and—when applicable—those of the ligands. k_{SC} – force constant employed to restrain side-chain heavy atoms. Δt – time step.

Step	Length [ns]	Δt [fs]	Restraints	k_{BB} [kJ mol^{-1} nm^{-2}]	k_{SC} [kJ mol^{-1} nm^{-2}]
1	1.0	1	heavy atoms	1000	500
2	1.0	1	heavy atoms	500	250
3	1.0	1	heavy atoms	250	100
4	2.0	2	heavy atoms	100	50
5	2.0	2	heavy atoms	50	0
6	0.5	2	none	0	0

Table S2: The average distances in Å between atoms forming hydrogen bonds HB1, HB2, and HB3, evaluated in REUS windows corresponding to the free-energy minima in T, R(transposed), and R(transition 2). Averages were calculated from the second halves of the trajectories; values in parentheses indicate standard deviations.

	**HB1**	**HB2**	**HB3**
**T**	2.11 (±0.23)	1.97 (±0.25)	2.75 (±0.06)
**R(transposed)**	2.24 (±0.74)	3.43 (±1.18)	6.05 (±2.04)
**R(transition 2)**	2.06 (±0.21)	1.97 (±0.19)	2.74 (±0.54)

HB1: R345:O–E767:HN; **HB2:** R345:HN–E765:O; **HB3:** S343:O–E765:HN.

Table S3: The average occupancy for hydrogen bonds HB1, HB2, and HB3, evaluated in REUS windows corresponding to the free-energy minima in T, R(transposed), and R(transition 2). Averages were calculated from the second halves of the trajectories; values in parentheses indicate SEM.

	**HB1**	**HB2**	**HB3**
**T**	0.94 (±0.01)	0.96 (±0.01)	0.42 (±0.02)
**R(transposed)**	0.88 (±0.01)	0.33 (±0.01)	0.01 (±0.00)
**R(transition 2)**	0.91 (±0.01)	0.95 (±0.01)	0.37 (±0.02)

HB1: R345:O–E767:HN; **HB2:** R345:HN–E765:O; **HB3:** S343:O–E765:HN.
